# Geraniol suppresses prostate cancer growth through down‐regulation of E2F8

**DOI:** 10.1002/cam4.864

**Published:** 2016-09-28

**Authors:** Sanghoon Lee, Yu Rang Park, Su‐Hwa Kim, Eun‐Jung Park, Min Ji Kang, Insuk So, Jung Nyeo Chun, Ju‐Hong Jeon

**Affiliations:** ^1^ Department of BiochemistryUniversity of Utah School of MedicineSalt Lake CityUtah84112 5650; ^2^ Office of Clinical Research InformationAsan Medical CenterSeoul05535Korea; ^3^ Department of Physiology and Biomedical SciencesSeoul National University College of MedicineSeoul03080Korea; ^4^ Department of Biomedical SciencesUniversity of Ulsan College of MedicineSeoul05535Korea; ^5^ Institute of Human‐Environment Interface BiologySeoul National UniversitySeoul03080Korea

**Keywords:** Bioinformatics, cell cycle control, clustering analysis, E2F8, geraniol, master regulator analysis, prostate cancer

## Abstract

Geraniol, an acyclic dietary monoterpene, has been found to suppress cancer survival and growth. However, the molecular mechanism underlying the antitumor action of geraniol has not been investigated at the genome‐wide level. In this study, we analyzed the microarray data obtained from geraniol‐treated prostate cancer cells. Geraniol potently altered a gene expression profile and primarily down‐regulated cell cycle‐related gene signatures, compared to linalool, another structurally similar monoterpene that induces no apparent phenotypic changes. Master regulator analysis using the prostate cancer‐specific regulatory interactome identified that the transcription factor E2F8 as a specific target molecule regulates geraniol‐specific cell cycle signatures. Subsequent experiments confirmed that geraniol down‐regulated E2F8 expression and the knockdown of E2F8 was sufficient to suppress cell growth by inducing G_2_/M arrest. Epidemiological analysis showed that E2F8 is up‐regulated in metastatic prostate cancer and associated with poor prognosis. These results indicate that E2F8 is a crucial transcription regulator controlling cell cycle and survival in prostate cancer cells. Therefore, our study provides insight into the role of E2F8 in prostate cancer biology and therapeutics.

## Introduction

Advanced or metastatic prostate cancer is initially amenable to androgen deprivation therapy (ADT), but eventually escapes from the therapeutic pressures and evolves into castration‐resistant prostate cancer (CRPC) 1. The molecular mechanisms of adaptive resistance to ADT have been extensively studied to identify new therapeutic options 2, 3. Various therapeutic agents against CRPC were approved 4, 5 or currently under evaluation in clinical trials 6. However, there are still no effective therapeutic regimens available for CRPC. Therefore, the targetable molecules and pathways for biological vulnerability of CRPC are needed to be identified to develop novel promising therapeutic modalities.

Geraniol is an acyclic monoterpene alcohol that is found in essential oils of fruits and herbs 7. Geraniol has been shown to exert a chemotherapeutic activity against various types of cancer, such as pancreas and colon cancer 8, 9, 10. We have found that geraniol specifically suppresses the growth of prostate cancer in cultured cell and xenograft tumor models 11, 12, compared to linalool, another structurally similar monoterpene that induce no apparent phenotype changes 12, 13. These results suggest that geraniol can be exploited as chemical moiety for developing anticancer drugs or as a valuable chemical probe for dissecting complex biological processes, discovering hidden molecular relations, and identifying therapeutic target molecules and pathways. However, the molecular mechanisms by which geraniol induces the changes in cellular phenotypes have been little investigated, particularly at the genome‐wide level.

In this study, we performed DNA microarray experiments to explore the molecular mechanisms of antitumor action of geraniol in prostate cancer cells. Through computational analyses, we identified that E2F8 is a target transcription factor of geraniol and the knockdown of E2F8 suppresses cell growth. In addition, we found that E2F8 is associated with poor survival of prostate cancer patients. Our findings provide insight into understanding the antitumor actions of geraniol, unraveling the role of E2F8 in CRPC biology, and developing novel therapeutic strategies.

## Materials and Methods

### Cell culture and microarray

PC‐3 prostate cancer cells were treated with 1 mmol/L geraniol or linalool in 0.1% ethanol (vehicle) for 24 h and then DNA microarray experiments were performed as described in our previous study 14. The microarray data are available through the Gene Expression Omnibus (GEO) database (accession number GSE45567). Rigorous data preprocessing for quality control and normalization were performed as described in our previous study 15. The combined microarray data set contained 11,877 genes which were mapped from microarray probes using a custom mapping file from the BrainArray resource (version 14.0.0) (http://brainarray.mbni.med.umich.edu/brainarray/). The normalized data of 121 microarray samples were used for constructing PC‐3 cell‐specific interactome, which is available from GEO (GSE67157).

### Unsupervised clustering analysis, internal cluster validation, and supervised classification

For the transcriptional subtyping of the microarray data, we performed unbiased clustering analysis in a uniform manner. We used an unsupervised feature selection method to determine 5000 genes of high variance across all microarray samples 16 and then performed clValid on the data of 5000 genes to measure an optimized clustering size using different clustering algorithms such as, Clustering LARge Applications (CLARA), hierarchical, k‐means 17, and Partitioning Around Medoids (PAM) 18. In clValid test, Dunn index for every cluster size each clustering algorithm was obtained to assess both intracluster homogeneity and intercluster separation by the ratio of the smallest distance between observations not in the same cluster to the largest intracluster distance 19. The clusters were validated through principal component analysis (PCA). The first and second principal components in the PCA plot were used to calculate the sum of % variances, and average (*λ*) and standard deviation (*σ*) of the Euclidean distance (ED) between a medoid and a sample in each cluster. For supervised classification, Random Forest, which is an ensemble learning method for classification and regression, was used to validate the clusters 20.

### SAM and GSEA

Significance analysis of microarrays (SAM) analysis was used to identify differentially expressed genes (DEGs) out of the 5000 genes among the clusters. A tuning parameter, delta of 0.4, optimized the cutoff for significance with the estimation of false discovery rate (FDR) threshold *q*‐value of 0.01. GSEA (gene set enrichment analysis) using geraniol‐specific DEGs was performed to obtain biological interpretation of gene signatures, and visualized mutually enriched gene signatures using EnrichmentMap 21.

### Prostate cancer‐specific interaction network and MRA

ARACNe algorithm 22 was used to reconstruct prostate cancer‐specific transcriptional interactome as previously described 15 with a few modifications. The 5000 genes of high variance in the microarray data and transcription factors (TFs) were used to infer the direct interactions among TFs and their regulons. We updated the list of human TFs from Animal Transcription Factor Database (AnimalTFDB) 23 and transcriptional regulatory gene signatures of Gene Ontology (GO), which are registered in MsigDB 4.0 24, such as REGULATION_OF_TRANSCRIPTION (GO:0045449) and TRANSCRIPTION_FACTOR_ACTIVITY (GO: 0003700). MRA‐Fisher's exact test (FET) 25 and MAster Regulator INference algorithm (MARINa) 26 were used to infer master regulator candidates and their targets in prostate cancer interactome. The ARACNe preprocessing and MRA‐FET analysis were run in geWorkbench software. MARINa results were visualized by geWorkbench version 2.6.0 (http://www.geworkbench.org) 27.

### Biomedical data analysis

We analyzed the microarray data sets of prostate cancer patients, GSE21034 28 and GSE3325 29, to examine E2F8 expression levels in different prostate cancer stages using ANOVA test. After normalization as described in our previous study 14, Affymetrix probe sets were mapped to Entrez Gene IDs using custom mapping files from the BrainArray resource (version 17.1.0). Then, the data sets of GSE21034 and GSE3325 were combined by overlapping genes and went through batch adjusting. Statistical analysis and data processing were performed using R (version 3.1.2) (https://www.r-project.org/) 30. The nonparametric Kaplan–Meier method was employed to obtain survival curves and the log‐rank test was used to determine overall survival from the International Cancer Genome Consortium (ICGC) dataset as previously described 31. For these analyses, *P = *0.05 was considered significant.

### Lab experiments

RT‐PCRs were performed using specific primers for E2F8 and *β*‐actin 32, 33. Antibody against E2F8 was obtained from Bethyl laboratories (Montgomery, TX). Antibodies to pCdk2^Y15^, pHistone H3^S10^, Cdk2, and Histone H3 were purchased from Abcam (Cambridge, UK). Anti‐tubulin antibody was supplied by Sigma‐Aldrich (St. Louis, MO). The cell extracts were resolved on 6–13.5% SDS‐PAGE gels and were probed with the indicated antibodies. Cell cycle and growth was assessed by propidium iodide staining and 3‐(4,5‐Dimethylthiazol‐2‐yl)‐2,5‐diphenyltetrazolium bromide (MTT) assays 15. Mitotic block experiments were performed as previously described 34. Cells were synchronized by arresting them at G_1_/S phase with 2 mmol/L thymidine 18 h, followed by a 4 h release, and then cells were arrested at G_2_/M phase with 100 ng/mL nocodazole for 16 h. Geraniol was treated at the same time as nocodazole. The cells were released from nocodazole block by washing with fresh medium. For a rescue experiment, PC‐3 cells were transfected with human E2F8 in pcDNA3 plus pEGFP and then incubated with 1 mmol/L geraniol for 24 h prior to flow cytometric analysis or western blotting.

### Statistical analysis

The comparison of the means among experimental groups was performed using one‐way ANOVA (Bonferroni's multiple comparison test) followed by a post hoc test. For these analyses, *P *= 0.05 was considered significant.

## Results

### Geraniol induces a distinctive gene expression profile

**Figure 1:**
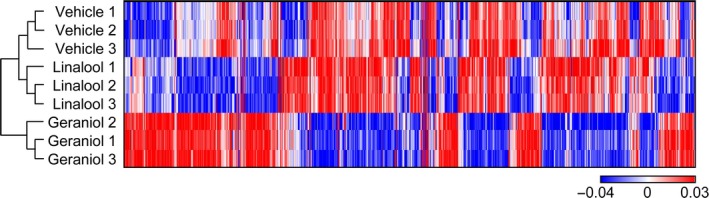
Geraniol alters a gene expression profile. Hierarchical clustering illustrates large‐scale differences in gene expression among vehicle‐, linalool‐, or geraniol‐treated PC‐3 cell samples.

We have previously shown that geraniol specifically suppresses cell survival and growth in PC‐3 cells, compared to linalool 11. To better understand the mechanism of action of geraniol, we compared gene expression profiles using the microarray data (GSE45567) obtained from vehicle‐, linalool‐, and geraniol‐treated PC‐3 cells. Our scheme for computational analyses is described in Figure S1. Hierarchical clustering algorithm clustered the microarray samples with whole 11,877 genes into three distinct groups (Fig. 1). These results indicate that geraniol induces the distinctive changes in gene expression, compared to linalool.

We then selected 5000 genes of the high variance across the nine samples, and confirmed that they still represent the same hierarchical clustering as Figure 1 (Fig. S2A). An optimal cluster size was estimated by clValid algorithm in various unsupervised clustering algorithms to assess cluster stability. clValid predicted that the cluster size 2 would be the most dependable by every clustering algorithm with the maximum Dunn index of 1.17 (Table S1). For validation, CLARA identified that vehicle‐ and linalool‐treated samples constructed one cluster and geraniol‐treated samples constituted another cluster by ED metric. However, internal validation by PCA and supervised classification by Random Forest showed that vehicle‐, linalool‐, and geraniol‐treated samples can be separated into independent clusters, supporting the hierarchical clustering analysis (Fig. S2B and Table S2). These findings reaffirm that geraniol induces a unique gene expression profile change.

### Geraniol regulates cell cycle gene signatures

**Figure 2:**
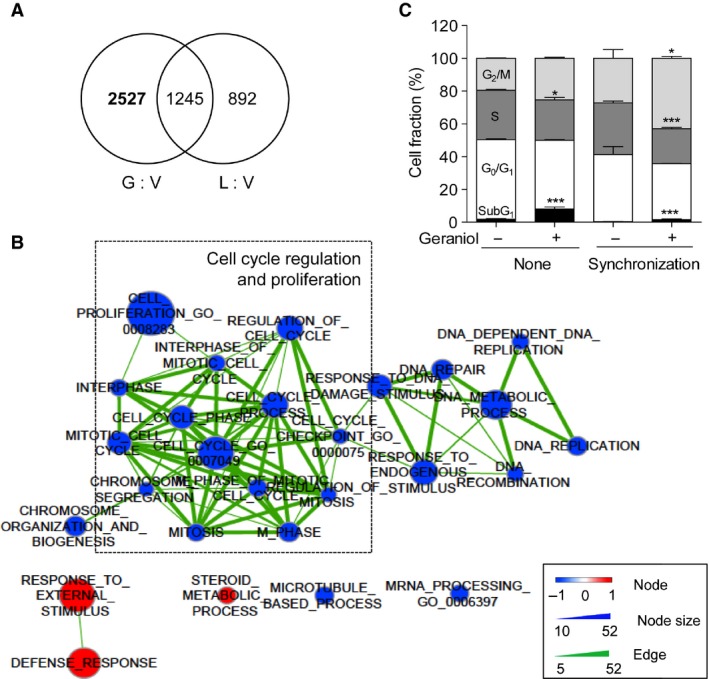
Geraniol down‐regulates cell cycle gene signatures. (A) Geraniol specifically alters the expression levels of 2527 genes. (B) Network visualization of GO enrichment analysis. Blue nodes represent down‐regulated and red nodes represent up‐regulated GO‐terms in the geraniol cluster. Node color intensity, node size, and edge thickness are proportional to value of normalized enrichment score (NES), the number of genes in gene signature, and the number of overlapping genes between two connected nodes. (C) PC‐3 cells were synchronized by thymidine‐nocodazole blockage and then treated with 1 mM linalool or geraniol for 24 h prior to flow cytometric analysis. Cell fraction is expressed as the percentage of cells in each phase of the cell cycle. The data were expressed as the mean ± SEM (*n* = 3). *P < 0.05, ***P < 0.005. V, vehicle; L, linalool, G, geraniol; GO, Gene Ontology.

SAM analysis revealed that geraniol specifically affected the expression levels of 2527 genes in PC‐3 cells (Fig. 2A). Using these genes, we examined the effect of geraniol on GO biological process. GSEA identified 25 down‐regulated and 3 up‐regulated gene signatures with *q*‐value 0.2 (Table S3). Of the 28 gene signatures, 13 signatures were associated with cell cycle or proliferation and 8 cell cycle‐related signatures were placed on the top with small normalized enrichment score (NES) values (indicated by blue in Table S3). The network visualization of enriched GO terms also showed that the geraniol primarily down‐regulates cell cycle‐ or proliferation‐related gene signatures (Fig. 2B). To confirm these computational findings, we analyzed cell cycle profile. Geraniol increased G_2_/M phase, which is more evident in thymidine‐nocodazole block experiments (Fig. 2C). Geraniol prevented the cell relief from nocodazole block.

### E2F8 is identified as a master regulator in prostate cancer

We found 79 leading edge subset (LES) genes (Table S4) overlapping across the 13 cell cycle‐related signatures and denoted them as geraniol‐specific cell cycle signatures for MRA. We then investigated the transcriptional regulatory mechanisms that determine the geraniol‐specific cell cycle signatures. ARACNe algorithm inferred a small consensus network of 161,446 interactions from the 462 human TF hub markers and the 5000 genes of high variance. From this transcriptional interactome, the MRA algorithm identified 40 TFs as master regulator (MR) candidates that down‐regulate geraniol‐specific cell cycle signatures. In addition, the MARINa algorithm inferred 224 TFs as MR candidates that differentiate the geraniol cluster from the vehicle one (Table S5): the 71 MRs out of 226 were negatively enriched and 153 MRs were positively enriched in the geraniol cluster.

**Figure 3:**
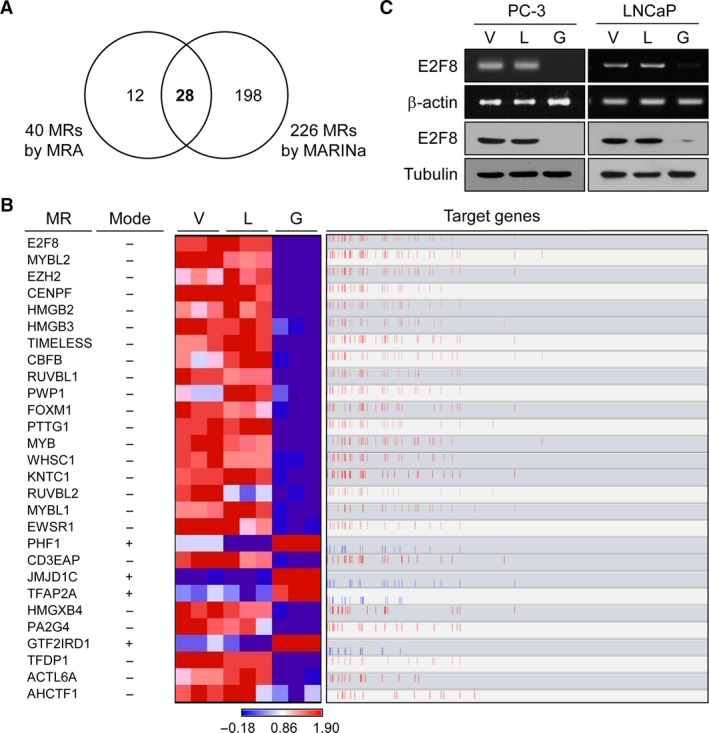
E2F8 as a master regulator controls geraniol‐specific cell cycle signatures. (A) 28 master regulators (MRs) are overlapping between the 40 MRs found by master regulator analysis and 226 MRs predicted by MARINa. (B) The heatmap shows the differences in gene expression levels of the 28 MRs in each sample. The mode explains whether geraniol positively (+) or negatively (−) affects the expression of MRs. Bar graph shows the distribution of positively (red) or negatively (blue) correlated target genes of the MRs (Spearman's correlation between the expression levels of the MR and its targets). (C) PC‐3 or LNCaP cells were treated with 1 mM linalool or geraniol for 24 h prior to RT‐PCR (upper) or western blot analysis (lower). *β*‐actin or tubulin was used as an internal or a loading control, respectively.

We then found that 28 MRs are commonly found in both the MRA and the MARINa algorithms (Fig. 3A and Table 1). The gene expression levels of 28 MRs were markedly altered in the geraniol cluster (heatmap in Fig. 3B) and correlated with those of their target genes (red bars in Fig. 3B). Of the 28 MRs, E2F8 as a top inactivated MR in the geraniol cluster was identified to regulate 58 target genes in the geraniol‐specific cell cycle signature genes (Table S6 and Fig. S3). Out of the 58 target genes, 18 genes are involved in G_2_/M phase cell cycle process according to molecular signature database (indicated in bold in Table S6). Indeed, geraniol specifically reduced the expression of E2F8 at both mRNA and protein levels in PC‐3, compared to linalool (Figs. 3C and S6). We also obtained the comparable results from LNCaP prostate cancer cells (Fig. 3C). In addition, we identified that geraniol regulated the expression levels of other several master regulators (Fig. S4).

**Table 1:** List of 28 master regulators that control geraniol‐specific cell cycle signatures

Gene ID	Gene symbol	Gene description	FET *P* ‐Value^a^	Markers in regulon^b^	Markers in intersection set^c^	Mode^d^	Odds Ratio^e^	NES^f^	absNES	Fold Change^g^	*q*‐value^g^
79733	E2F8	E2F transcription factor 8	3.31E‐43	478	58	—	15.00	−13.72	13.72	0.66	0.00
4605	MYBL2	v‐myb myeloblastosis viral oncogene homolog‐like 2	9.02E‐30	539	50	—	15.77	−13.79	13.79	0.68	0.00
2146	EZH2	Enhancer of zeste homolog 2 (Drosophila)	7.32E‐24	578	46	—	12.25	−14.68	14.68	0.73	0.00
1063t	CENPF	Centromere protein F, 350/400 kDa (mitosin)	1.75E‐21	493	41	—	16.32	−12.51	12.51	0.56	0.00
3148	HMGB2	High mobility group box 2	3.34E‐20	501	40	—	8.74	−12.44	12.44	0.63	0.00
3149t	HMGB3	High mobility group box 3	6.28E‐24	400	40	—	16.41	−11.34	11.34	0.75	0.00
8914	TIMELESS	Timeless homolog (Drosophila)	1.04E‐21	457	40	—	10.64	−12.38	12.38	0.83	0.00
865	CBFB	Core‐binding factor, beta subunit	8.91E‐15	597	37	—	9.97	−13.25	13.25	0.85	0.01
8607	RUVBL1	RuvB‐like 1 (*E. coli*)	1.29E‐11	751	37	—	9.14	−13.41	13.41	0.73	0.00
11137	PWP1	PWP1 homolog (*S. cerevisiae*)	1.47E‐14	571	36	—	6.98	−12.81	12.81	0.90	0.24
2305	FOXM1	Forkhead box M1	2.07E‐15	504	35	—	11.99	−13.21	13.21	0.69	0.00
9232	PTTG1	Pituitary tumor‐transforming 1	2.52E‐15	475	34	—	10.12	−12.19	12.19	0.54	0.00
4602	MYB	v‐myb myeloblastosis viral oncogene homolog	4.96E‐14	524	34	—	11.80	−11.35	11.35	0.62	0.00
7468	WHSC1	Wolf‐Hirschhorn syndrome candidate 1	9.73E‐12	590	33	—	9.17	−13.29	13.29	0.77	0.00
9735	KNTC1	Kinetochore associated 1	2.44E‐16	354	31	—	10.77	−10.71	10.71	0.70	0.00
10856	RUVBL2	RuvB‐like 2 (*E. coli*)	4.11E‐12	502	31	—	8.55	−11.67	11.67	0.83	0.00
4603	MYBL1	v‐myb myeloblastosis viral oncogene homolog‐like 1	4.01E‐14	336	28	—	11.84	−10.73	10.73	0.76	0.00
2130	EWSR1	Ewing sarcoma breakpoint region 1	5.32E‐13	372	28	—	12.31	−8.79	8.79	0.82	0.00
5252	PHF1	PHD finger protein 1	1.46E‐10	468	28	+	7.60	11.45	11.45	1.16	0.02
10849	CD3EAP	CD3e molecule, epsilon‐associated protein	3.76E‐11	378	26	—	17.60	−11.50	11.50	0.79	0.00
221037	JMJD1C	Jumonji domain containing 1C	1.08E‐08	453	25	+	19.28	9.36	9.36	1.32	0.00
7020	TFAP2A	Transcription factor AP‐2 alpha	4.85E‐10	359	24	+	5.16	6.01	6.01	1.23	0.00
10042	HMGXB4	HMG box domain containing 4	1.86E‐05	582	23	—	7.59	−10.37	10.37	0.76	0.00
5036	PA2G4	Proliferation‐associated 2G4, 38 kDa	2.06E‐08	364	22	—	14.26	−11.18	11.18	0.82	0.00
9569	GTF2IRD1	GTF2I repeat domain containing 1	1.07E‐07	331	20	+	6.67	7.26	7.26	1.19	0.01
7027	TFDP1	Transcription factor Dp‐1	2.12E‐06	398	20	—	15.56	−11.73	11.73	0.70	0.00
86	ACTL6A	Actin‐like 6A	1.55E‐06	287	17	—	7.17	−3.26	3.26	0.76	0.00
25909	AHCTF1	AT hook containing transcription factor 1	1.96E‐05	346	17	—	7.02	−8.44	8.44	0.83	0.01

The 28 MRs were sorted by largest to smallest by Markers in intersection set in descendent order. Odds ratio, NES, fold change and *q*‐value were rounded off to two decimal places.

a FET *P ‐*value, the *P ‐*value from Fisher's exact test. It shows how much significantly the marker (gene) belongs to the signature set and the regulon of the master regulator (MR).

b Markers in regulon, the number of markers (genes) found to be first neighbors of the master regulator in the loaded network.

c Makers in intersection set, the number of markers found in the intersection of the signature and the regulon of the candidate MR.

d Mode, plus mode means that the MR is positively correlated with up‐regulated regulons in geraniol cluster, and minus mode means that the MR is positively correlated with down‐regulated genes in geraniol cluster.

e Odd Ratio, odds of a regulon gene being in the GSEA leading edge set/odds of a regulon gene being in the GSEA trailing edge set.

f NES, GSEA normalized enrichment score for the regulon of the TF.

g Fold change and *q‐value* were calculated from SAM analysis.

### E2F8 knockdown induces G_2_/M arrest

**Figure 4:**
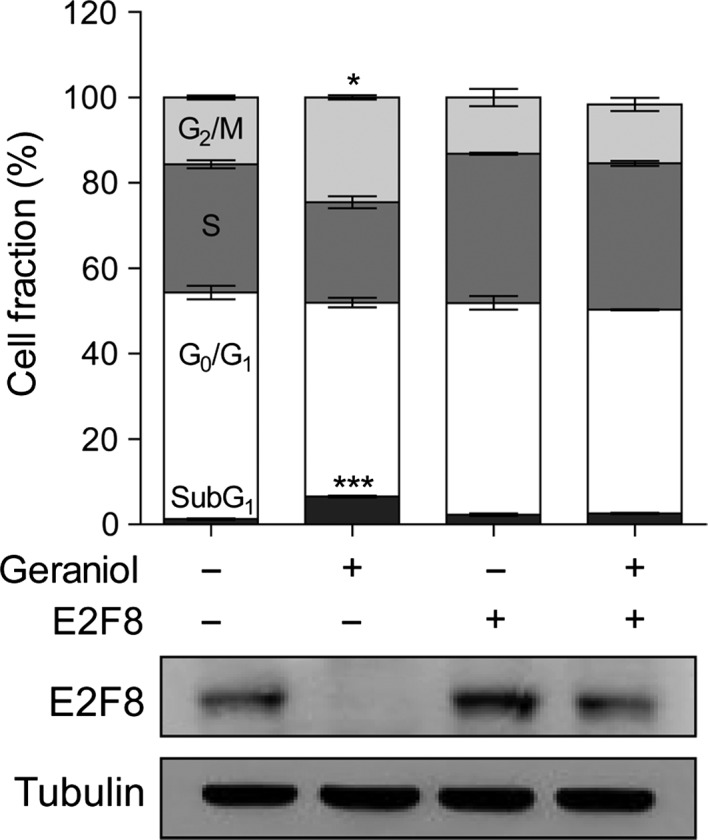
The effect of E2F8 overexpression on geraniol‐induced G_2_/M arrest. PC‐3 cells were transfected with human E2F8 in pcDNA3 and then incubated with 1 mmol/L geraniol for 24 h prior to flow cytometric analysis (upper) or western blotting (lower). *P < 0.05, ***P < 0.005.

To assess the role of E2F8 in geraniol‐induced phenotypic changes, rescue experiments were performed. We found that E2F8 overexpression reversed geraniol‐induced G_2_/M arrest (Fig. 4). We then knocked down the level of E2F8 using siRNA against E2F8 (siE2F8). We first tested three different siE2F8s in PC‐3 cells. All tested siE2F8s suppressed cell growth and induced G_2_/M arrest (Fig. S5), excluding the off‐target effect of siRNAs. Because siE2F8‐1 was more effective than other siE2F8s, we chose siE2F8‐1 in following studies.

**Figure 5:**
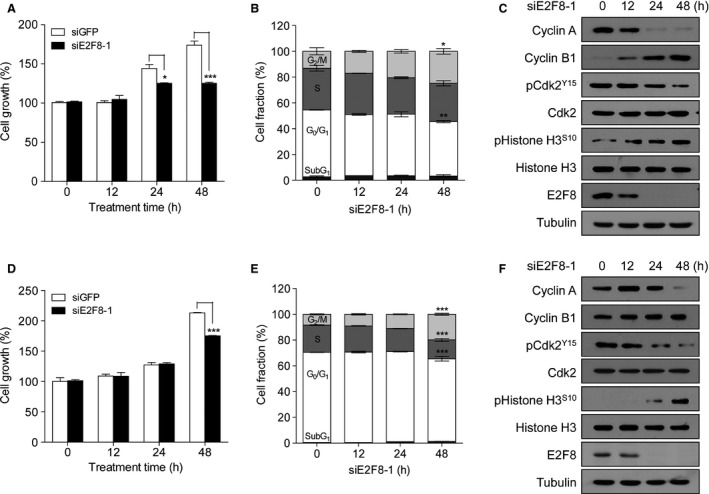
E2F8 knockdown induces G_2_/M arrest and apoptosis. PC‐3 (A–C) or LNCaP (D–F) cells were transfected with 100 nmol/L siE2F8 for the indicated times prior to MTT assay (A and D), cell cycle analysis (B and E), and western blot analysis (C and F). Cell growth was expressed as a relative value compared to that of siGFP as a control which was set to 100%. The data were expressed as the mean ± SEM (*n* = 3). *P < 0.05, **P < 0.01, ***P < 0.005.

We examined the effect of siE2F8‐1 on cell cycle profile in PC‐3 and LNCaP cells. MTT assays showed that E2F8 knockdown suppressed cell growth (Fig. 5A and D). In addition, siE2F8‐1 markedly increased cell populations in the G_2_/M phase (Fig. 5B and E) and induced the changes in the levels of cyclin A, cyclin B1, phospho‐CDK2, and phospho‐histone H3, which are G_2_/M transition regulatory proteins (Fig. 5C and F). These results indicate that E2F8 knockdown substantially reproduces geraniol‐induced G_2_/M arrest phenotype. In addition, our findings suggest that E2F8 plays a crucial role in the regulation of G_2_/M cell cycle in prostate cancer cells.

### Clinical relevance of E2F8 in prostate cancer

**Figure 6:**
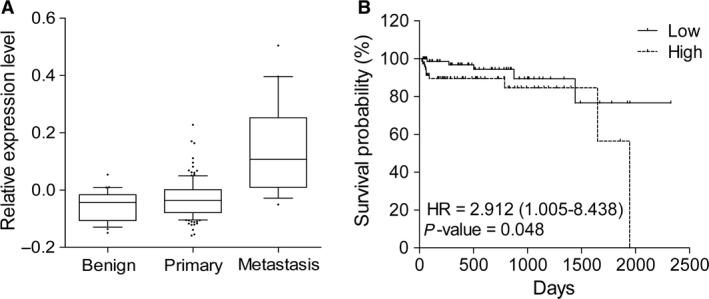
Clinical relevance of E2F8 in prostate cancer patients. (A) The expression levels of E2F8 in prostate cancer patient samples (GSE21034 and GSE3325) were represented in the box‐plot. The *x*‐axis indicates three different stages of prostate cancer and *y*‐axis represents the normalized expression level of E2F8. (B) Survival curve for prostate cancer patients (ICGC dataset) based on the expression levels of E2F8. HR, hazard ratio (95% confidence interval).

To address the clinical relevance of our findings, we examined the expression levels of E2F8 using microarray datasets of prostate cancer patients (GSE21034 and GSE3325) 28, 29. The averages of normalized E2F8 expression levels were calculated from benign, primary, and metastatic tumor samples; its relative expression levels were ‐0.054, −0.025, and 0.146, respectively (ANOVA test, *P *= 3.44e^−12^; Fig. 6A). This result suggests that the increased expression of E2F8 is associated with prostate cancer metastasis. To assess the effect of E2F8 on clinical outcome, we compared overall survival in the prostate cancer patients with low‐ and high‐expression of E2F8. Kaplan–Meier analysis indicated that the patients with high expression of E2F8 had significantly worse overall survival than those with low expression (HR = 2.912 and log‐rank test *P *= 0.048) (Fig. 6B).

## Discussion

In this study, we described four main findings: (1) geraniol specifically induces the changes in cell cycle gene signatures; (2) geraniol reduces the expression levels of the transcription factor E2F8; (3) E2F8 controls G_2_/M cell cycle progression; and (4) E2F8 is up‐regulated in metastatic prostate cancer and associated with worse survival in prostate cancer patients.

We have found that geraniol can be a useful chemical probe for dissecting the complicated phenotypes of prostate cancer and identifying therapeutic target molecules 10, 11. In this study, we performed clustering analysis, pathway‐based approach, and master regulator analysis using the microarray data and identified E2F8 as a target TF of geraniol. E2F8 is a member of atypical E2F family that regulates cell survival and embryonic development 35. However, it is unclear whether E2F8 functions as a transcriptional repressor or activator 36, 37. Emerging evidence suggests that E2F8 contributes to the oncogenic potentials of several types of cancer, such as hepatic or lung cancer 30, 31. Our study reveals that E2F8 exerts pro‐oncogenic activity in prostate cancer via G_2_/M cell cycle regulation.

Our results indicate that E2F8 is required for the growth of prostate cancer cells and is aberrantly expressed in metastatic prostate cancer. In addition, we demonstrate that the overexpression of E2F8 has clinically relevant prognostic significance in prostate cancer. Therefore, these findings suggest that E2F8 is a novel targetable molecule for treatment of metastatic prostate cancer or CRPC patients. Our results provide a basis for future investigations aiming at elucidating the molecular mechanisms underlying the expression or activity of E2F8 in prostate cancer, which assists to understand the role of E2F8 in CRPC biology and to develop novel cell cycle‐targeted therapeutic strategies.

## Conflict of Interest

We hereby declare that we have no pecuniary or other personal interest, direct or indirect, in any matter that raises a conflict.

## Supporting information


**Figure S1**. Schematic diagram for the computational approaches.

**Figure S2**. Gene expression profiles and PCA plot.

**Figure S3.** Gene expression of E2F8 target genes across three clusters.

**Figure S4.** The expression levels of master regulators.

**Figure S5.** The effect of siRNAs against E2F8 on cell growth and cell cycle.

**Figure S6.** Full scan images of used in this study.

**Table S1.** Summary of Dunn index in the different cluster size by clValid analysis.
**Table S2.** Classification of monoterpene‐treated PC‐3 cell microarray samples by Random Forest.
**Table S3**. List of 28 gene signatures of Gene Ontology Biological Process in the geraniol cluster.
**Table S4.** List of 79 LES genes that cover all of the LES genes from cell cycle and proliferation gene signatures.
**Table S5**. The result of MARINa analysis: 224 TFs that were activated or inactive in the geraniol cluster.
**Table S6**. List of 58 target genes of E2F8.
