# Supplementary figures and images for: Geraniol suppresses prostate cancer growth through down‐regulation of E2F8
Source: Cancer Med. 2016 Sep 28;5(10):2899–908. doi: 10.1002/cam4.864 (PMC5083744; doi:10.1002/cam4.864)

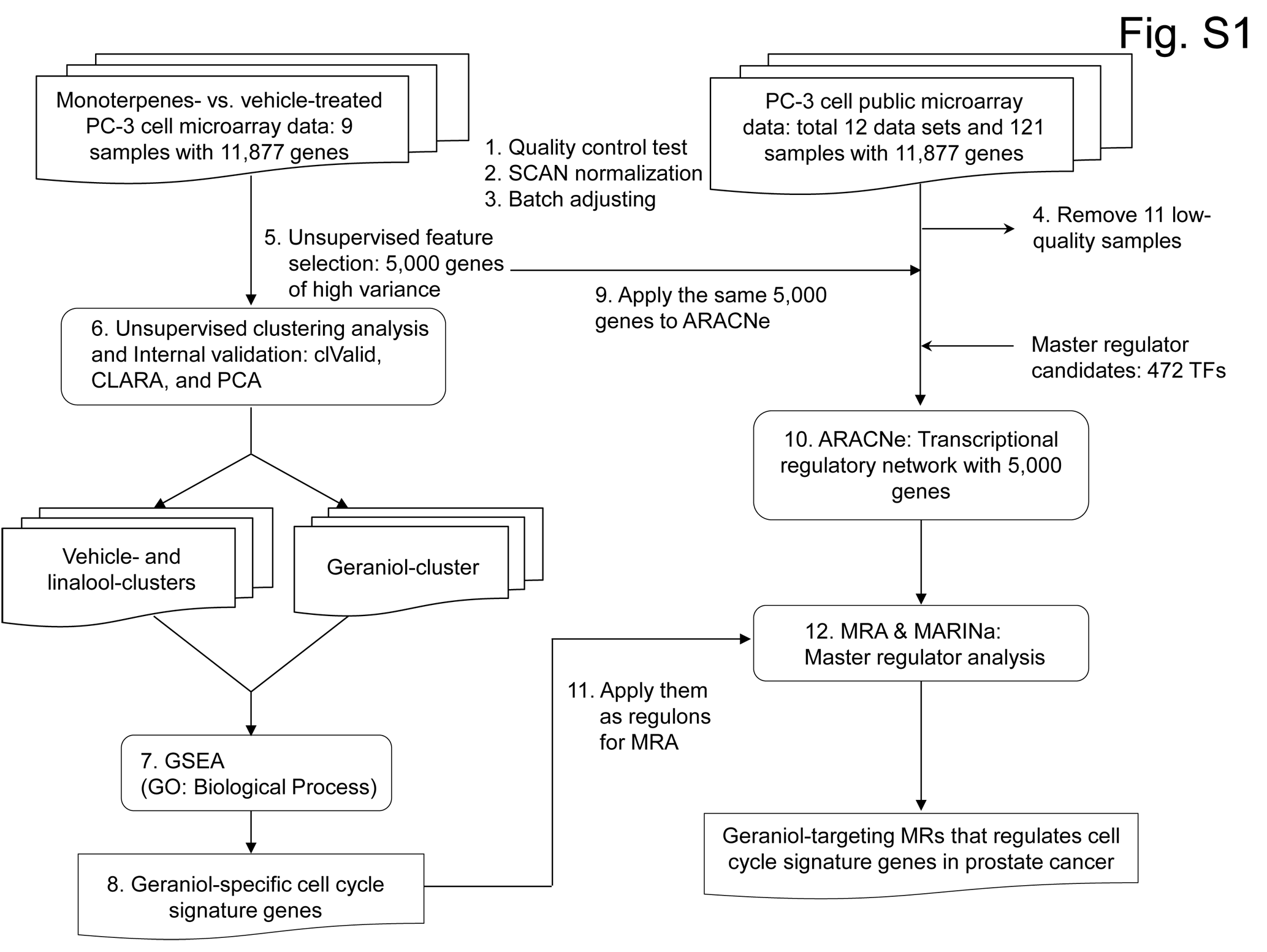

Supplement: Supplementary file 1 — Figure S1. Schematic diagram for the computational approaches. [file CAM4-5-2899-s001.tiff]

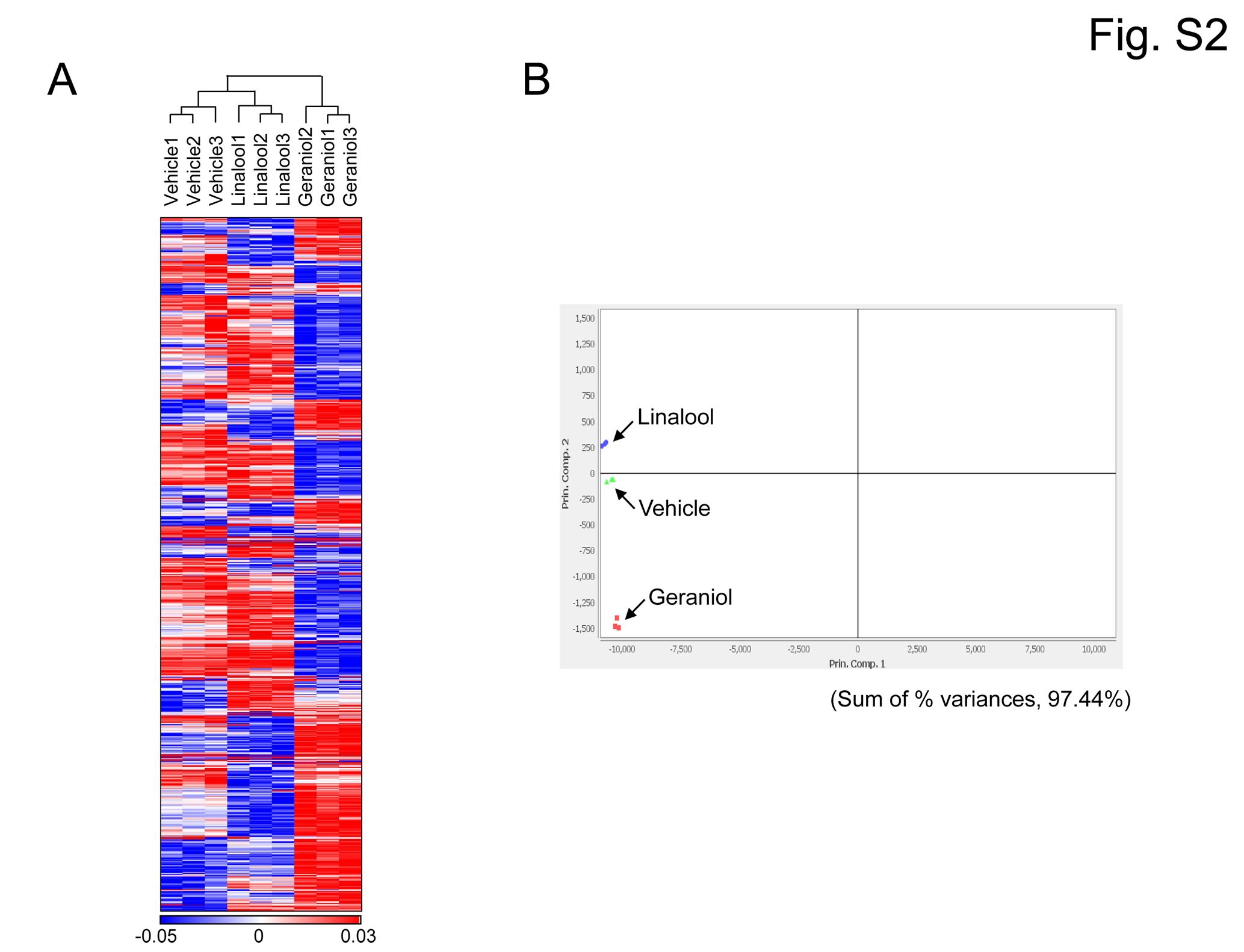

Supplement: Supplementary file 2 — Figure S2. Gene expression profiles and PCA plot. [file CAM4-5-2899-s002.tiff]

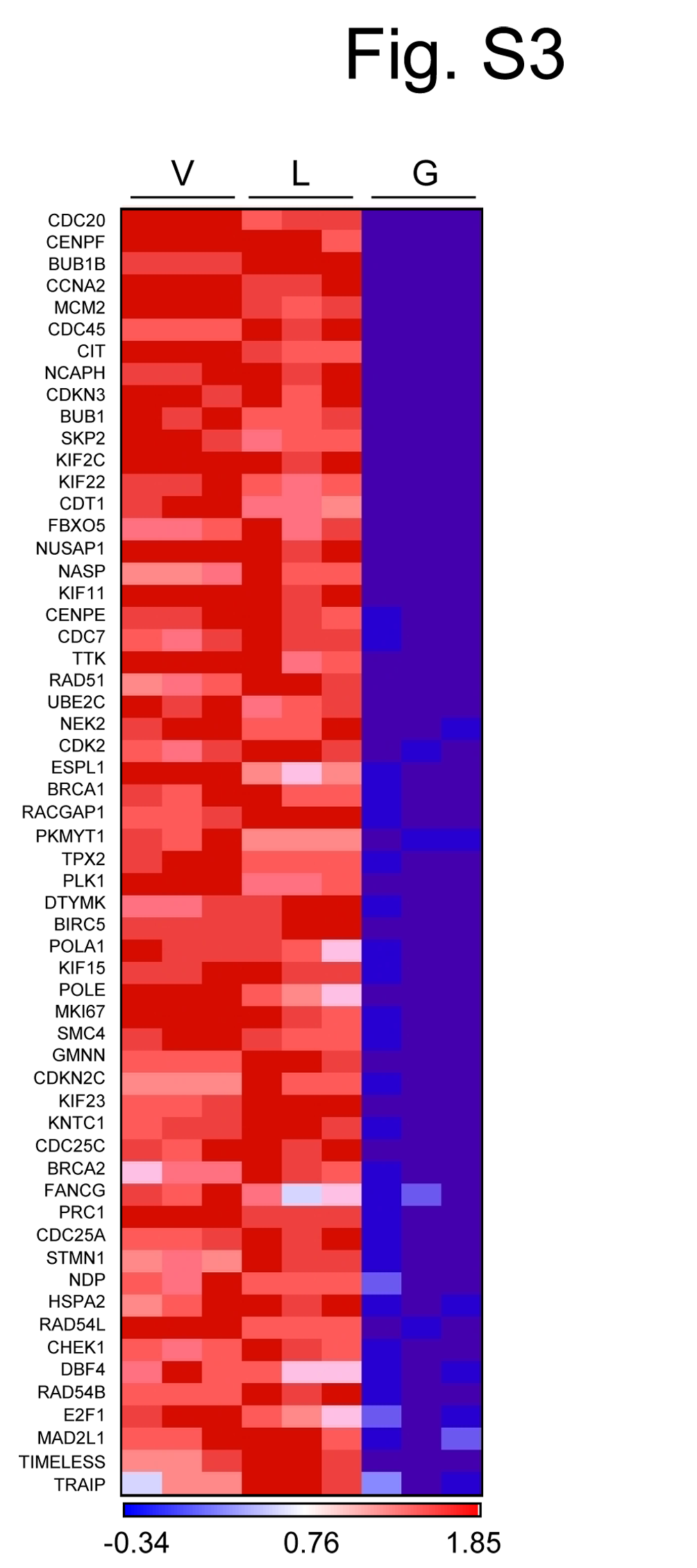

Supplement: Supplementary file 3 — Figure S3. Gene expression of E2F8 target genes across three clusters. [file CAM4-5-2899-s003.tiff]

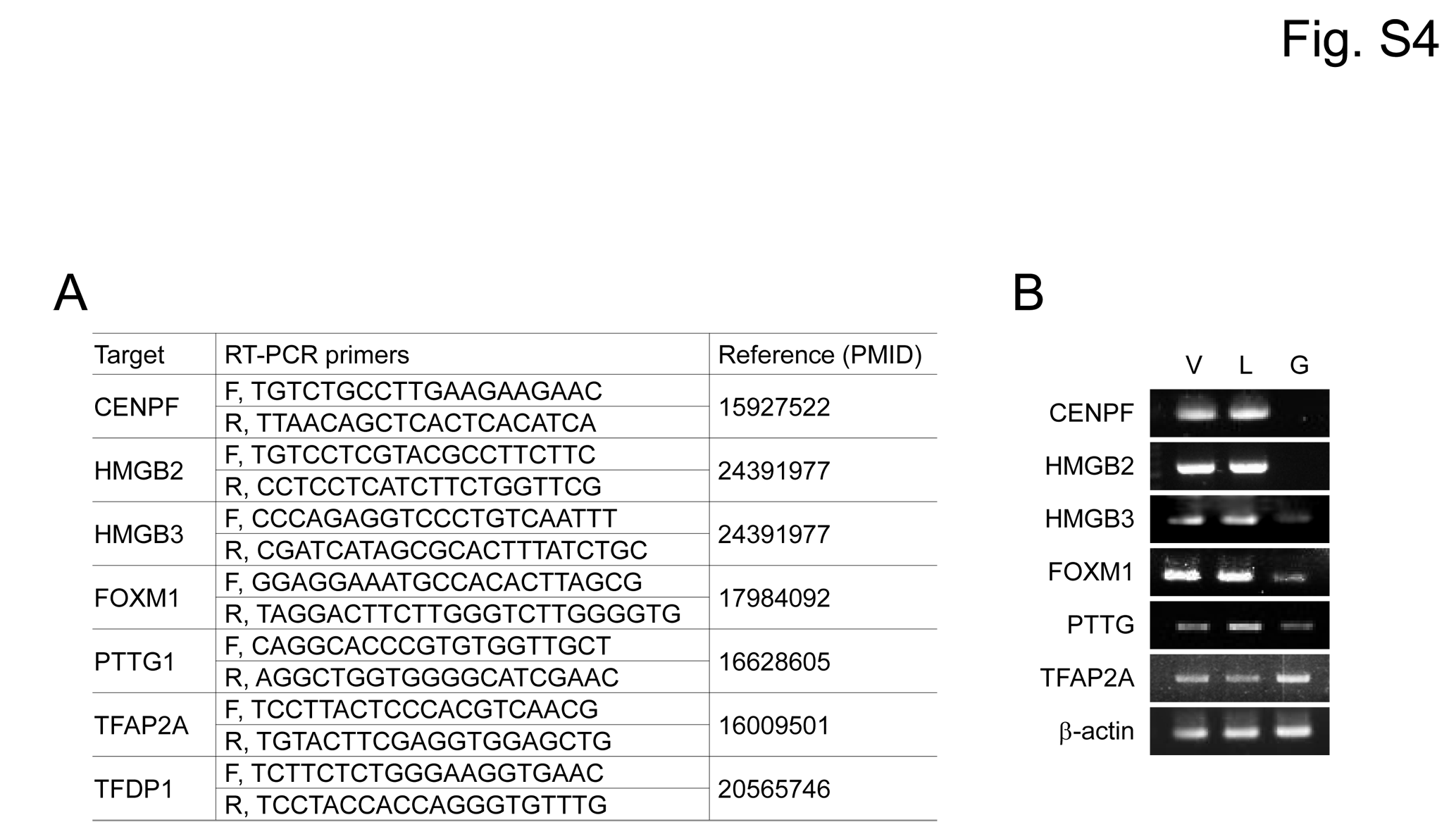

Supplement: Supplementary file 4 — Figure S4. The expression levels of master regulators. [file CAM4-5-2899-s004.tiff]

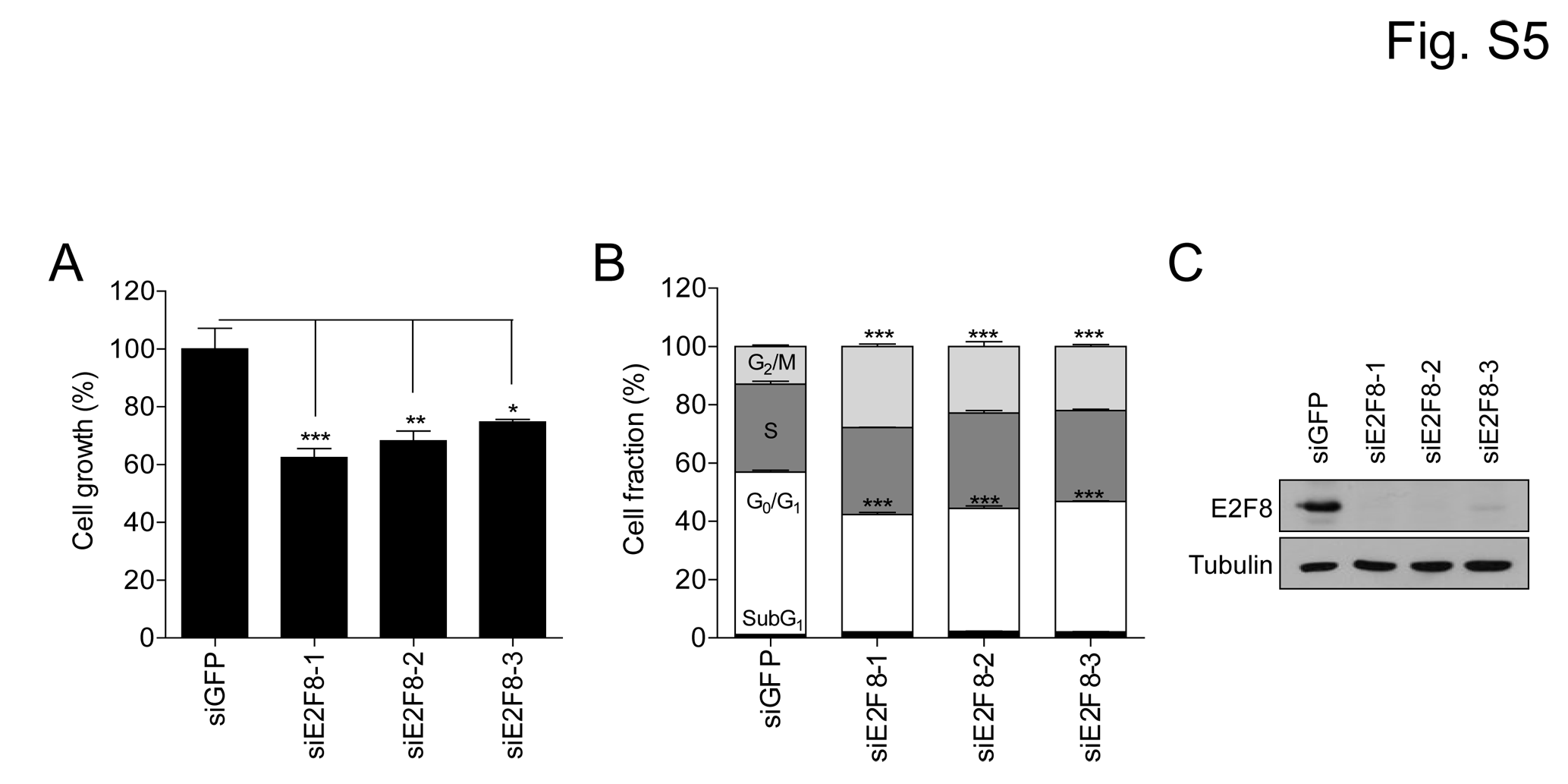

Supplement: Supplementary file 5 — Figure S5. The effect of siRNAs against E2F8 on cell growth and cell cycle. [file CAM4-5-2899-s005.tiff]

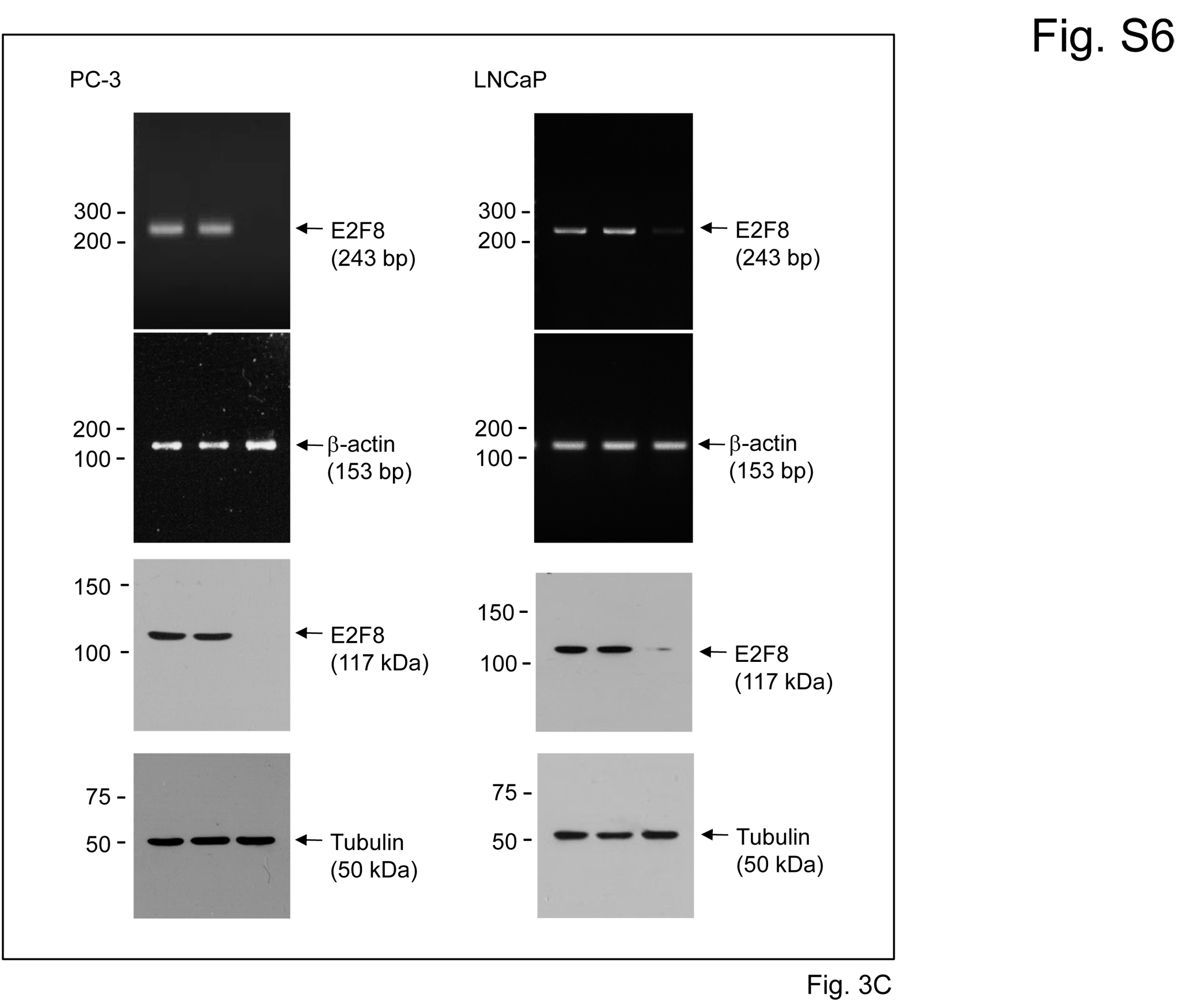

Supplement: Supplementary file 6 — Figure S6. Full scan images of used in this study. [file CAM4-5-2899-s006.tiff]

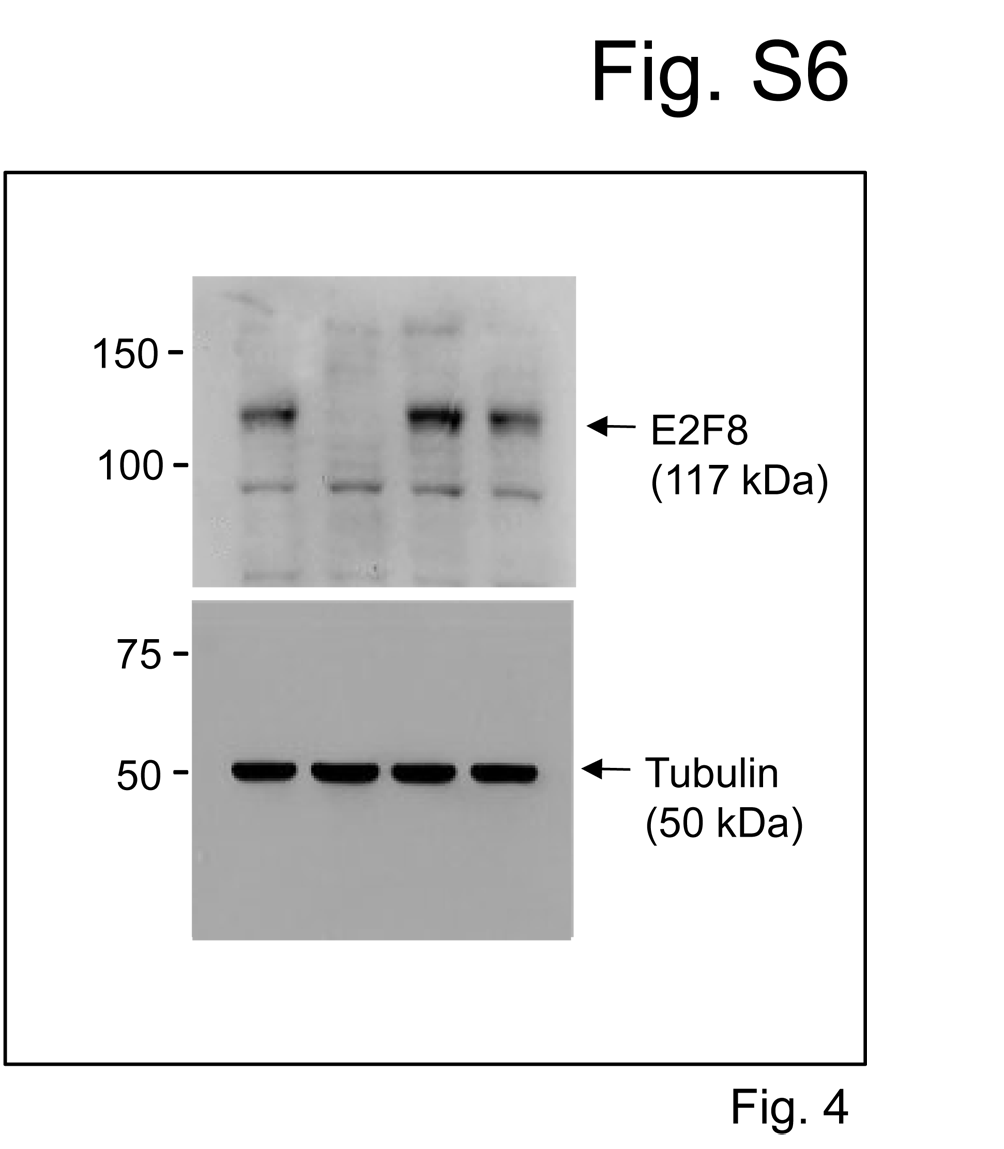

Supplement: Supplementary file 7 [file CAM4-5-2899-s007.tiff]

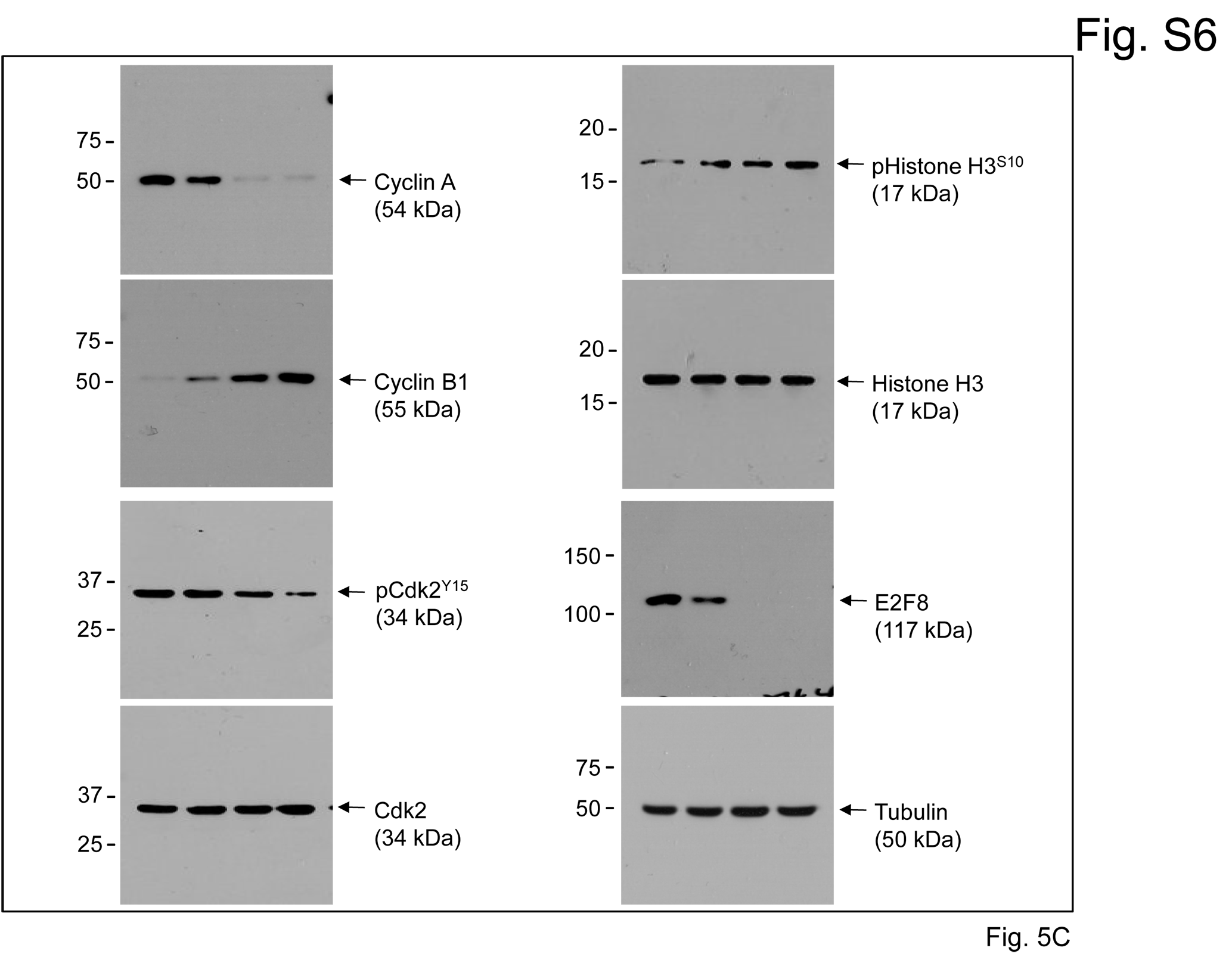

Supplement: Supplementary file 8 [file CAM4-5-2899-s008.tiff]

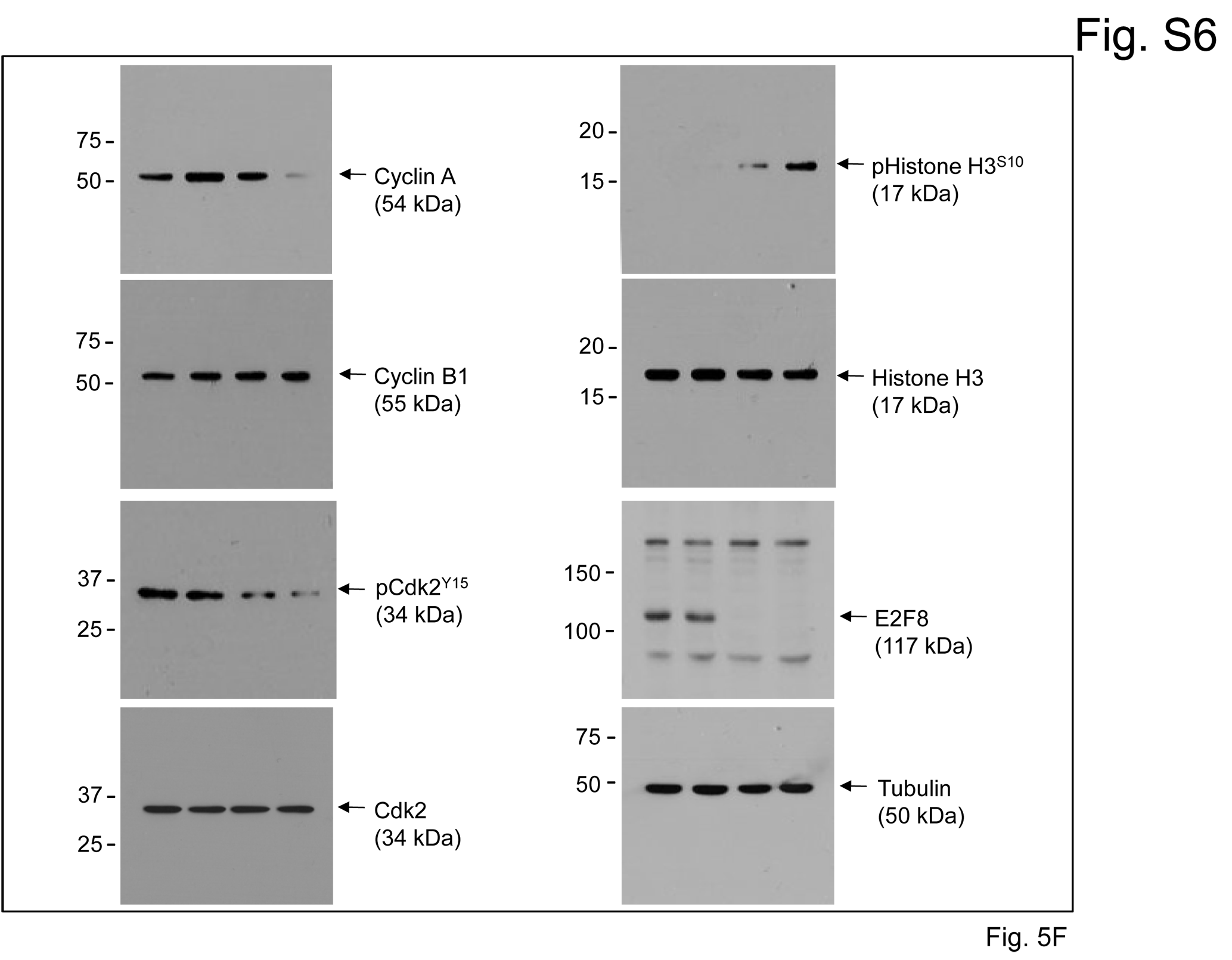

Supplement: Supplementary file 9 [file CAM4-5-2899-s009.tiff]

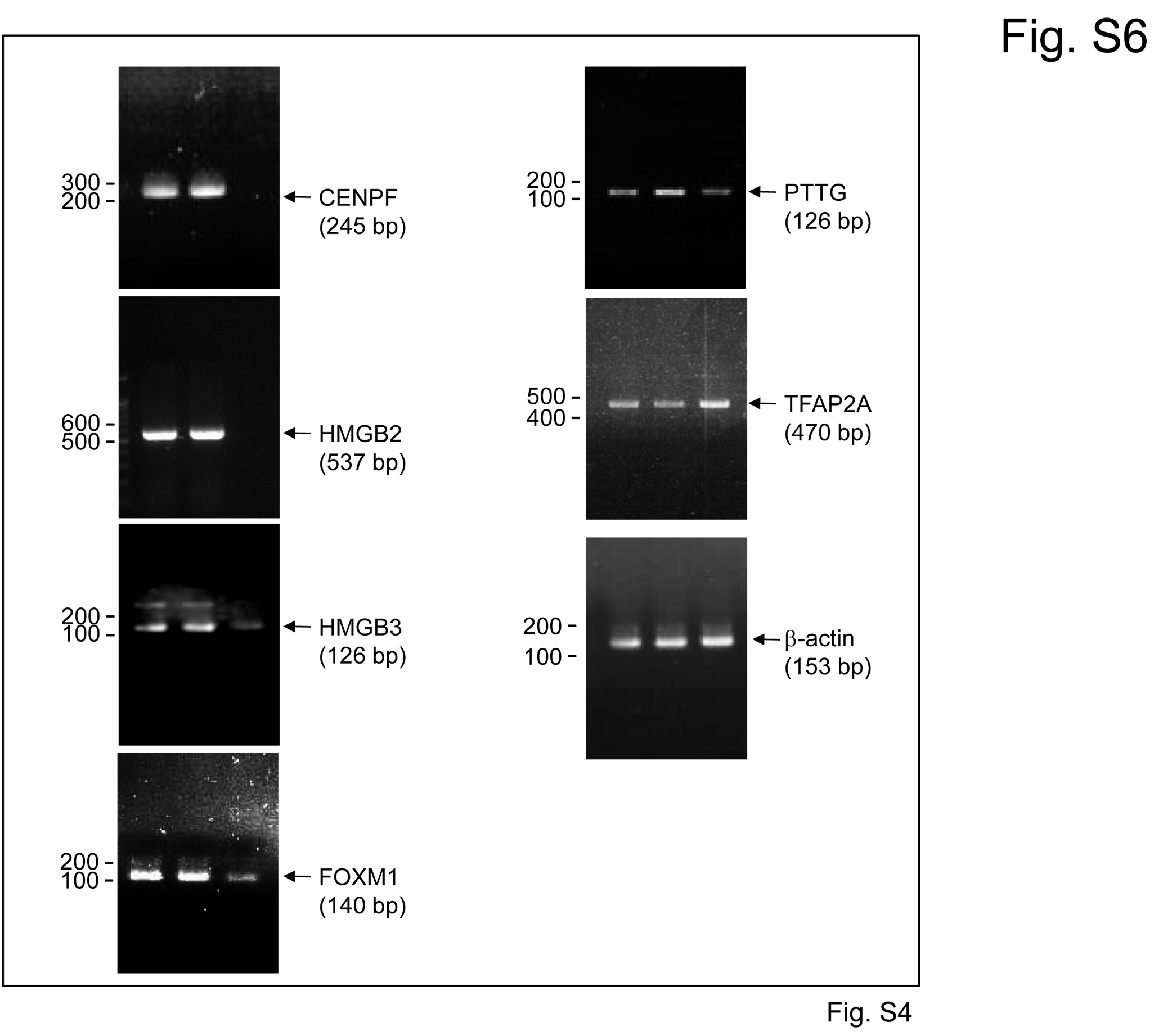

Supplement: Supplementary file 10 [file CAM4-5-2899-s010.tiff]

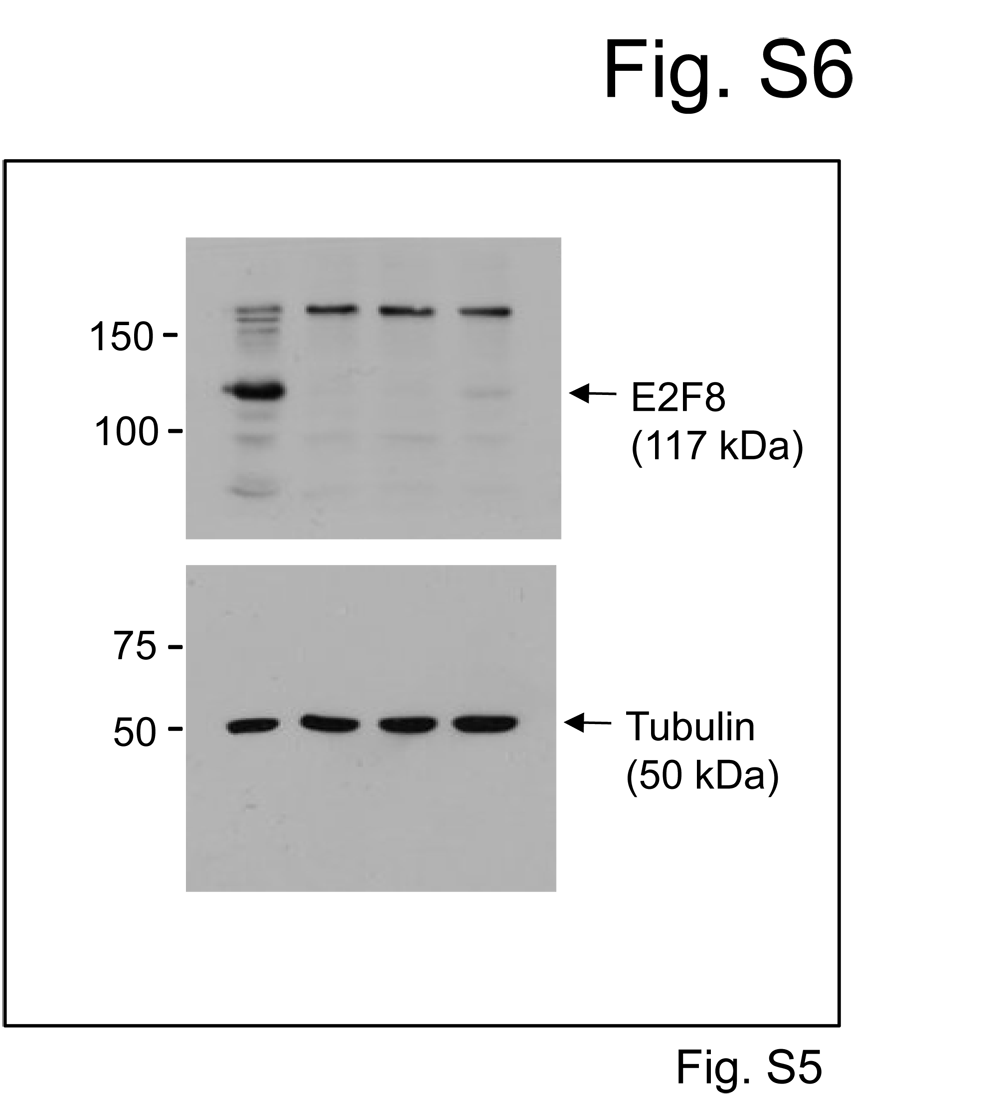

Supplement: Supplementary file 11 [file CAM4-5-2899-s011.tiff]

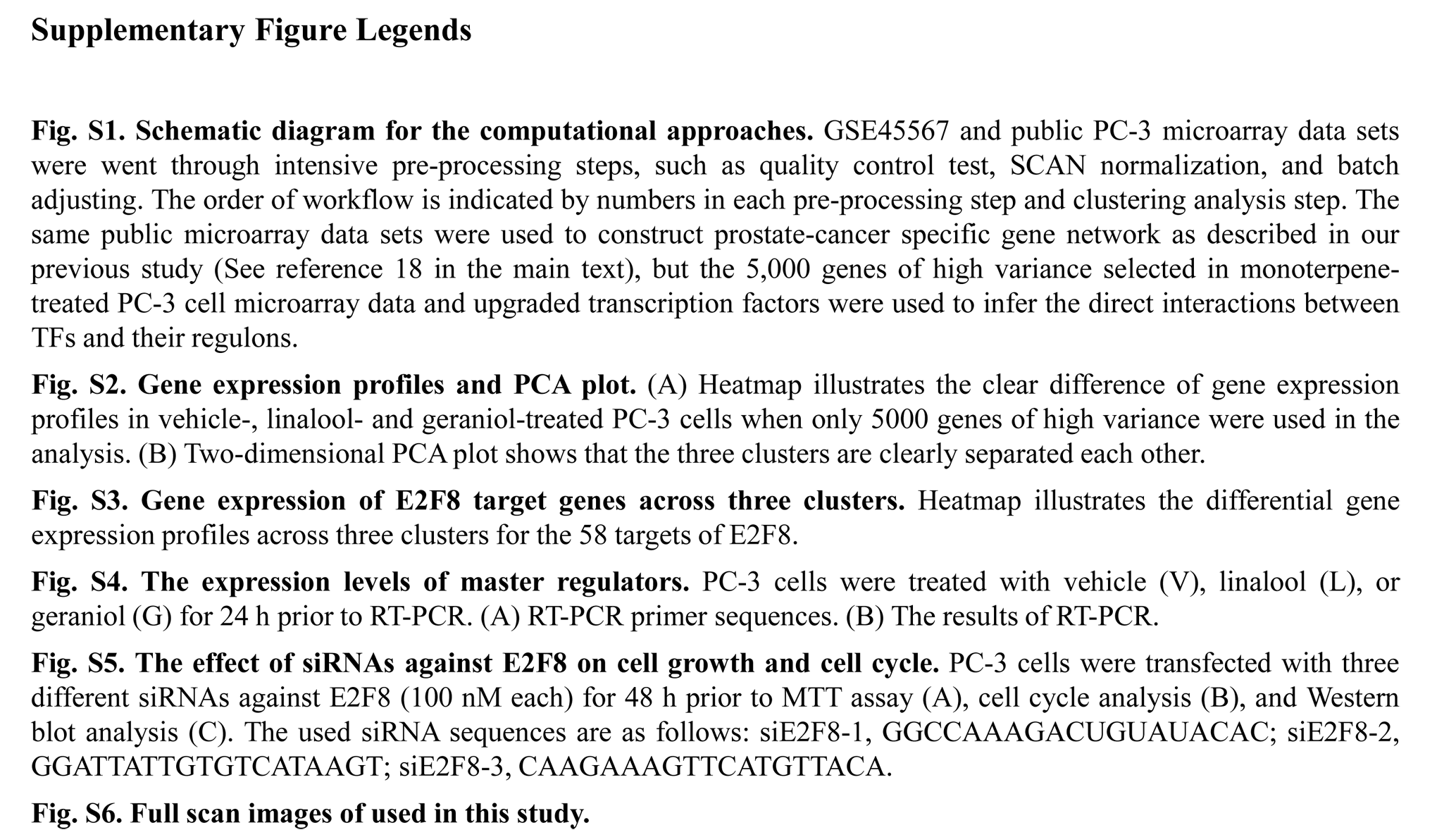

Supplement: Supplementary file 14 [file CAM4-5-2899-s014.tiff]
