# Supplementary material for: Geraniol suppresses prostate cancer growth through down‐regulation of E2F8
Source: Cancer Med. 2016 Sep 28;5(10):2899–908. doi: 10.1002/cam4.864 (PMC5083744; doi:10.1002/cam4.864)
Supplement: Supplementary file 12 — Table S1. Summary of Dunn index in the different cluster size by clValid analysis. Table S2. Classification of monoterpene‐treated PC‐3 cell microarray samples by Random Forest. Table S3. List of 28 gene signatures of Gene Ontology Biological Process in the geraniol cluster. Table S4. List of 79 LES genes that cover all of the LES genes from cell cycle and proliferation gene signatures. Table S5. The result of MARINa analysis: 224 TFs that were activated or inactive in the geraniol cluster. Table S6. List of 58 target genes of E2F8. [file CAM4-5-2899-s012.docx]

| **Table S1.** Summary of Dunn index in the different cluster size by clValid analysis. | | | | |
| --- | --- | --- | --- | --- |
|  | *size 2* | *size 3* | *size 4* | *size 5* |
| CLARA | **1.17** | 1.01 | 0.78 | 1.15 |
| Hierarchical | **1.17** | 1.01 | 0.78 | 1.15 |
| k-means | **1.17** | 1.01 | 0.78 | 1.15 |
| PAM | **1.17** | 1.01 | 0.78 | 1.15 |

| **Table S2.** Classification of monoterpene-treated PC-3 cell microarray samples by Random Forest. | | | | |  | |
| --- | --- | --- | --- | --- | --- | --- |
| SampleID | ActualClass | PredictedClass | Vehicle_Probability | Linalool_Probability | | Geraniol_Probability |
| GSM1109801.CEL | Vehicle | Vehicle | **0.73** | 0.17 | | 0.10 |
| GSM1109802.CEL | Vehicle | Vehicle | **0.77** | 0.16 | | 0.07 |
| GSM1109803.CEL | Vehicle | Vehicle | **0.68** | 0.20 | | 0.12 |
| GSM1109816.CEL | Linalool | Linalool | 0.21 | **0.72** | | 0.07 |
| GSM1109817.CEL | Linalool | Linalool | 0.21 | **0.74** | | 0.05 |
| GSM1109818.CEL | Linalool | Linalool | 0.22 | **0.74** | | 0.04 |
| GSM1109810.CEL | Geraniol | Geraniol | 0.13 | 0.07 | | **0.79** |
| GSM1109811.CEL | Geraniol | Geraniol | 0.12 | 0.08 | | **0.80** |
| GSM1109812.CEL | Geraniol | Geraniol | 0.11 | 0.09 | | **0.80** |

**Table S3**. List of 28 gene signatures of Gene Ontology Biological Process in the geraniol cluster.

| No. | Name | Mode ^a^ | ES ^b^ | NES ^b^ | Nominal *p-value* | FDR *q-value* ^c^ |
| --- | --- | --- | --- | --- | --- | --- |
| 1 | **CELL_CYCLE_PROCESS** | **-** | -0.67 | -2.83 | 0.00 | 0.00 |
| 2 | **M_PHASE** | **-** | -0.72 | -2.78 | 0.00 | 0.00 |
| 3 | **CELL_CYCLE_PHASE** | **-** | -0.67 | -2.77 | 0.00 | 0.00 |
| 4 | **M_PHASE_OF_MITOTIC_CELL_CYCLE** | **-** | -0.74 | -2.74 | 0.00 | 0.00 |
| 5 | **MITOSIS** | **-** | -0.74 | -2.72 | 0.00 | 0.00 |
| 6 | **MITOTIC_CELL_CYCLE** | **-** | -0.67 | -2.69 | 0.00 | 0.00 |
| 7 | **CELL_CYCLE_GO_0007049** | **-** | -0.56 | -2.52 | 0.00 | 0.00 |
| 8 | **REGULATION_OF_MITOSIS** | **-** | -0.74 | -2.28 | 0.00 | 0.00 |
| 9 | CHROMOSOME_SEGREGATION | - | -0.76 | -2.19 | 0.00 | 0.00 |
| 10 | DNA_METABOLIC_PROCESS | - | -0.51 | -2.15 | 0.00 | 0.00 |
| 11 | DNA_REPAIR | - | -0.54 | -2.10 | 0.00 | 0.00 |
| 12 | DNA_DEPENDENT_DNA_REPLICATION | - | -0.62 | -2.05 | 0.00 | 0.00 |
| 13 | DNA_RECOMBINATION | - | -0.67 | -2.03 | 0.00 | 0.00 |
| 14 | RESPONSE_TO_DNA_DAMAGE_STIMULUS | - | -0.50 | -2.01 | 0.00 | 0.00 |
| 15 | DNA_REPLICATION | - | -0.53 | -1.99 | 0.00 | 0.00 |
| 16 | **CELL_CYCLE_CHECKPOINT_GO_0000075** | **-** | -0.63 | -1.95 | 0.00 | 0.00 |
| 17 | **REGULATION_OF_CELL_CYCLE** | **-** | -0.47 | -1.87 | 0.00 | 0.01 |
| 18 | RESPONSE_TO_ENDOGENOUS_STIMULUS | - | -0.46 | -1.87 | 0.00 | 0.01 |
| 19 | CHROMOSOME_ORGANIZATION_AND_BIOGENESIS | - | -0.52 | -1.85 | 0.00 | 0.01 |
| 20 | **INTERPHASE** | **-** | -0.55 | -1.82 | 0.00 | 0.01 |
| 21 | **INTERPHASE_OF_MITOTIC_CELL_CYCLE** | **-** | -0.55 | -1.81 | 0.00 | 0.01 |
| 22 | MICROTUBULE_BASED_PROCESS | - | -0.51 | -1.73 | 0.01 | 0.02 |
| 23 | **CELL_PROLIFERATION_GO_0008283** | **-** | -0.36 | -1.58 | 0.00 | 0.08 |
| 24 | MRNA_PROCESSING_GO_0006397 | - | -0.47 | -1.55 | 0.03 | 0.09 |
| 25 | REGULATION_OF_DNA_METABOLIC_PROCESS | - | -0.50 | -1.48 | 0.06 | 0.15 |
| 26 | RESPONSE_TO_EXTERNAL_STIMULUS | + | 0.46 | 1.80 | 0.01 | 0.19 |
| 27 | DEFENSE_RESPONSE | + | 0.58 | 2.18 | 0.00 | 0.00 |
| 28 | STEROID_METABOLIC_PROCESS | + | 0.67 | 2.18 | 0.00 | 0.01 |
| Note: The values of ES, NES, Nominal *p-*value, and FDR *q-*value were rounded off to two decimal places. The table is sorted smallest to largest by NES.  ^a^ Plus mode means that the MR is positively correlated with up-regulated regulons in the geraniol-cluster, whereas minus mode denotes that the MR is positively correlated with down-regulated genes.  ^b^ The normalized enrichment score (NES) for the gene set is calculated by normalizing the enrichment score (ES) across analyzed gene sets.  ^c^ FDR corresponding to each NES is the estimated probability of false positives. FDR was represented as *q-*value, which is the analog of *p*-value that has been corrected for multiple hypothesis testing. | | | | | | |

| **Table S4.** List of 79 LES genes that cover all of the LES genes from cell-cycle and proliferation gene signatures | | | | | |
| --- | --- | --- | --- | --- | --- |
| No. | Gene ID | Gene symbol | Gene description | Fold change ^a^ | *q-value* ^a^ |
| 1 | 51203 | NUSAP1 | nucleolar and spindle associated protein 1 | 0.50 | 0.00 |
| 2 | 11065 | UBE2C | ubiquitin-conjugating enzyme E2C | 0.53 | 0.00 |
| 3 | 9055 | PRC1 | protein regulator of cytokinesis 1 | 0.53 | 0.00 |
| 4 | 9787 | DLGAP5 | discs, large (Drosophila) homolog-associated protein 5 | 0.54 | 0.00 |
| 5 | 51053 | GMNN | geminin, DNA replication inhibitor | 0.56 | 0.00 |
| 6 | 1063 | CENPF | centromere protein F, 350/400kDa (mitosin) | 0.56 | 0.00 |
| 7 | 29127t | RACGAP1 | Rac GTPase activating protein 1 | 0.57 | 0.00 |
| 8 | 22974 | TPX2 | TPX2, microtubule-associated, homolog (Xenopus laevis) | 0.57 | 0.00 |
| 9 | 4171 | MCM2 | minichromosome maintenance complex component 2 | 0.57 | 0.00 |
| 10 | 10051 | SMC4 | structural maintenance of chromosomes 4 | 0.57 | 0.00 |
| 11 | 1033 | CDKN3 | cyclin-dependent kinase inhibitor 3 | 0.58 | 0.00 |
| 12 | 7272 | TTK | TTK protein kinase | 0.59 | 0.00 |
| 13 | 6790 | AURKA | aurora kinase A | 0.60 | 0.00 |
| 14 | 701 | BUB1B | budding uninhibited by benzimidazoles 1 homolog beta (yeast) | 0.61 | 0.00 |
| 15 | 991 | CDC20 | cell division cycle 20 homolog (S. cerevisiae) | 0.61 | 0.00 |
| 16 | 56992 | KIF15 | kinesin family member 15 | 0.63 | 0.00 |
| 17 | 3832 | KIF11 | kinesin family member 11 | 0.63 | 0.00 |
| 18 | 11004 | KIF2C | kinesin family member 2C | 0.63 | 0.00 |
| 19 | 26298 | EHF | ets homologous factor | 0.64 | 0.00 |
| 20 | 9493 | KIF23 | kinesin family member 23 | 0.64 | 0.00 |
| 21 | 890 | CCNA2 | cyclin A2 | 0.64 | 0.00 |
| 22 | 3838 | KPNA2 | karyopherin alpha 2 (RAG cohort 1, importin alpha 1) | 0.64 | 0.00 |
| 23 | 4085 | MAD2L1 | MAD2 mitotic arrest deficient-like 1 (yeast) | 0.64 | 0.00 |
| 24 | 332 | BIRC5 | baculoviral IAP repeat containing 5 | 0.65 | 0.00 |
| 25 | 4288 | MKI67 | antigen identified by monoclonal antibody Ki-67 | 0.65 | 0.00 |
| 26 | 1164 | CKS2 | CDC28 protein kinase regulatory subunit 2 | 0.65 | 0.00 |
| 27 | 8317 | CDC7 | cell division cycle 7 homolog (S. cerevisiae) | 0.66 | 0.00 |
| 28 | 1062 | CENPE | centromere protein E, 312kDa | 0.67 | 0.00 |
| 29 | 11113 | CIT | citron (rho-interacting, serine/threonine kinase 21) | 0.67 | 0.00 |
| 30 | 5347 | PLK1 | polo-like kinase 1 | 0.67 | 0.00 |
| 31 | 9700 | ESPL1 | extra spindle pole bodies homolog 1 (S. cerevisiae) | 0.68 | 0.00 |
| 32 | 8438 | RAD54L | RAD54-like (S. cerevisiae) | 0.69 | 0.00 |
| 33 | 23397 | NCAPH | non-SMC condensin I complex, subunit H | 0.69 | 0.00 |
| 34 | 8318 | CDC45 | cell division cycle 45 homolog (S. cerevisiae) | 0.69 | 0.00 |
| 35 | 9735 | KNTC1 | kinetochore associated 1 | 0.70 | 0.00 |
| 36 | 1031 | CDKN2C | cyclin-dependent kinase inhibitor 2C (p18, inhibits CDK4) | 0.70 | 0.00 |
| 37 | 2013 | EMP2 | epithelial membrane protein 2 | 0.70 | 0.00 |
| 38 | 7516 | XRCC2 | X-ray repair complementing defective repair in Chinese hamster cells 2 | 0.70 | 0.00 |
| 39 | 26271 | FBXO5 | F-box protein 5 | 0.71 | 0.00 |
| 40 | 5764 | PTN | pleiotrophin | 0.71 | 0.00 |
| 41 | 699 | BUB1 | budding uninhibited by benzimidazoles 1 homolog (yeast) | 0.71 | 0.00 |
| 42 | 3553 | IL1B | interleukin 1, beta | 0.71 | 0.00 |
| 43 | 5422 | POLA1 | polymerase (DNA directed), alpha 1, catalytic subunit | 0.72 | 0.00 |
| 44 | 5608 | MAP2K6 | mitogen-activated protein kinase kinase 6 | 0.72 | 0.00 |
| 45 | 6502 | SKP2 | S-phase kinase-associated protein 2 (p45) | 0.73 | 0.00 |
| 46 | 1950 | EGF | epidermal growth factor | 0.74 | 0.00 |
| 47 | 81620 | CDT1 | chromatin licensing and DNA replication factor 1 | 0.74 | 0.00 |
| 48 | 25788 | RAD54B | RAD54 homolog B (S. cerevisiae) | 0.74 | 0.00 |
| 49 | 10198 | MPHOSPH9 | M-phase phosphoprotein 9 | 0.74 | 0.00 |
| 50 | 3835 | KIF22 | kinesin family member 22 | 0.75 | 0.00 |
| 51 | 7048 | TGFBR2 | transforming growth factor, beta receptor II (70/80kDa) | 0.75 | 0.00 |
| 52 | 1111 | CHEK1 | CHK1 checkpoint homolog (S. pombe) | 0.75 | 0.00 |
| 53 | 10926 | DBF4 | DBF4 homolog (S. cerevisiae) | 0.75 | 0.00 |
| 54 | 4693 | NDP | Norrie disease (pseudoglioma) | 0.75 | 0.00 |
| 55 | 4751 | NEK2 | NIMA (never in mitosis gene a)-related kinase 2 | 0.75 | 0.00 |
| 56 | 3306 | HSPA2 | heat shock 70kDa protein 2 | 0.75 | 0.00 |
| 57 | 672 | BRCA1 | breast cancer 1, early onset | 0.76 | 0.00 |
| 58 | 545 | ATR | ataxia telangiectasia and Rad3 related | 0.76 | 0.00 |
| 59 | 993 | CDC25A | cell division cycle 25 homolog A (S. pombe) | 0.76 | 0.00 |
| 60 | 995 | CDC25C | cell division cycle 25 homolog C (S. pombe) | 0.77 | 0.00 |
| 61 | 4678 | NASP | nuclear autoantigenic sperm protein (histone-binding) | 0.77 | 0.00 |
| 62 | 1017 | CDK2 | cyclin-dependent kinase 2 | 0.78 | 0.00 |
| 63 | 9088 | PKMYT1 | protein kinase, membrane associated tyrosine/threonine 1 | 0.78 | 0.00 |
| 64 | 2189 | FANCG | Fanconi anemia, complementation group G | 0.78 | 0.00 |
| 65 | 1663 | DDX11 | DEAD/H (Asp-Glu-Ala-Asp/His) box polypeptide 11 | 0.78 | 0.00 |
| 66 | 5424 | POLD1 | polymerase (DNA directed), delta 1, catalytic subunit 125kDa | 0.79 | 0.00 |
| 67 | 1841 | DTYMK | deoxythymidylate kinase (thymidylate kinase) | 0.80 | 0.00 |
| 68 | 5426 | POLE | polymerase (DNA directed), epsilon | 0.80 | 0.00 |
| 69 | 1869 | E2F1 | E2F transcription factor 1 | 0.81 | 0.00 |
| 70 | 5888 | RAD51 | RAD51 homolog (S. cerevisiae) | 0.82 | 0.00 |
| 71 | 3925 | STMN1 | stathmin 1 | 0.82 | 0.00 |
| 72 | 54962 | TIPIN | TIMELESS interacting protein | 0.83 | 0.00 |
| 73 | 1021 | CDK6 | cyclin-dependent kinase 6 | 0.83 | 0.00 |
| 74 | 8792 | TNFRSF11A | tumor necrosis factor receptor superfamily, member 11a, NFKB activator | 0.83 | 0.00 |
| 75 | 8914 | TIMELESS | timeless homolog (Drosophila) | 0.83 | 0.00 |
| 76 | 675 | BRCA2 | breast cancer 2, early onset | 0.84 | 0.00 |
| 77 | 7042 | TGFB2 | transforming growth factor, beta 2 | 0.85 | 0.01 |
| 78 | 5536 | PPP5C | protein phosphatase 5, catalytic subunit | 0.87 | 0.02 |
| 79 | 10293 | TRAIP | TRAF interacting protein | 0.89 | 0.11 |
| Note: The values of fold change and *q-*value were rounded off to two decimal places. The table is sorted smallest to largest by fold change.  ^a^ Fold change and *q-*value were calculated by SAM analysis. | | | | |  |
|  |  |  |  |  |  |

| **Table S5**. The result of MARINa analysis: 224 TFs that were activated or inactive in the geraniol cluster. | | | | | | | | | | |
| --- | --- | --- | --- | --- | --- | --- | --- | --- | --- | --- |
| No. | Gene ID | Gene symbol | Gene description | GSEA *p-value* | Markers in regulon ^a^ | Number Leading Edge ^b^ | Odd Ratio ^c^ | NES ^d^ | absolute NES | Mode ^e^ |
| 1 | 8607 | RUVBL1 | RuvB-like 1 (E. coli) | 0.00E+00 | 695 | 320 | 9.14 | -13.41 | 13.41 | - |
| 2 | 7468 | WHSC1 | Wolf-Hirschhorn syndrome candidate 1 | 0.00E+00 | 492 | 240 | 9.17 | -13.29 | 13.29 | - |
| 3 | 2146 | EZH2 | enhancer of zeste homolog 2 (Drosophila) | 0.00E+00 | 491 | 279 | 12.25 | -14.68 | 14.68 | - |
| 4 | 865 | CBFB | core-binding factor, beta subunit | 0.00E+00 | 480 | 223 | 9.97 | -13.25 | 13.25 | - |
| 5 | 11137 | PWP1 | TJSTODSL | 0.00E+00 | 477 | 206 | 6.98 | -12.81 | 12.81 | - |
| 6 | 4601 | MXI1 | MAX interactor 1 | 0.00E+00 | 465 | 163 | 3.96 | 10.50 | 10.50 | + |
| 7 | 4602 | MYB | v-myb myeloblastosis viral oncogene homolog (avian) | 0.00E+00 | 460 | 239 | 11.80 | -11.35 | 11.35 | - |
| 8 | 10042 | HMGXB4 | HMG box domain containing 4 | 0.00E+00 | 458 | 170 | 7.59 | -10.37 | 10.37 | - |
| 9 | 4605 | MYBL2 | v-myb myeloblastosis viral oncogene homolog (avian)-like 2 | 0.00E+00 | 449 | 233 | 15.77 | -13.79 | 13.79 | - |
| 10 | 3148 | HMGB2 | high mobility group box 2 | 0.00E+00 | 446 | 193 | 8.74 | -12.44 | 12.44 | - |
| 11 | 10481 | HOXB13 | homeobox B13 | 0.00E+00 | 443 | 72 | 8.05 | -7.22 | 7.22 | - |
| 12 | 79733 | **E2F8** | **E2F transcription factor 8** | **0.00E+00** | **437** | **236** | **15.00** | **-13.72** | **13.72** | **-** |
| 13 | 79618 | HMBOX1 | homeobox containing 1 | 0.00E+00 | 433 | 214 | 6.04 | 10.65 | 10.65 | + |
| 14 | 23085 | ERC1 | ELKS/RAB6-interacting/CAST family member 1 | 0.00E+00 | 428 | 131 | 4.57 | 8.75 | 8.75 | + |
| 15 | 3170 | FOXA2 | forkhead box A2 | 0.00E+00 | 427 | 112 | 9.77 | -8.44 | 8.44 | - |
| 16 | 2305 | FOXM1 | forkhead box M1 | 0.00E+00 | 427 | 235 | 11.99 | -13.21 | 13.21 | - |
| 17 | 3182 | HNRNPAB | heterogeneous nuclear ribonucleoprotein A/B | 0.00E+00 | 423 | 261 | 8.04 | -13.48 | 13.48 | - |
| 18 | 2965 | GTF2H1 | general transcription factor IIH, polypeptide 1, 62kDa | 0.00E+00 | 412 | 162 | 3.94 | 8.26 | 8.26 | + |
| 19 | 6304 | SATB1 | SATB homeobox 1 | 0.00E+00 | 407 | 91 | 6.54 | 8.62 | 8.62 | + |
| 20 | 2971 | GTF3A | general transcription factor IIIA | 0.00E+00 | 404 | 142 | 3.96 | -7.35 | 7.35 | - |
| 21 | 1063 | CENPF | centromere protein F, 350/400kDa (mitosin) | 0.00E+00 | 402 | 200 | 16.32 | -12.51 | 12.51 | - |
| 22 | 5252 | PHF1 | PHD finger protein 1 | 0.00E+00 | 402 | 183 | 7.60 | 11.45 | 11.45 | + |
| 23 | 3664 | IRF6 | interferon regulatory factor 6 | 0.00E+00 | 395 | 140 | 6.57 | 8.64 | 8.64 | + |
| 24 | 26298 | EHF | ets homologous factor | 0.00E+00 | 393 | 61 | 3.84 | -5.25 | 5.25 | - |
| 25 | 22882 | ZHX2 | zinc fingers and homeoboxes 2 | 0.00E+00 | 389 | 189 | 12.61 | 9.95 | 9.95 | + |
| 26 | 10468 | FST | follistatin | 0.00E+00 | 388 | 36 | 9.12 | 4.29 | 4.29 | + |
| 27 | 7024 | TFCP2 | transcription factor CP2 | 0.00E+00 | 388 | 92 | 1.52 | -4.50 | 4.50 | - |
| 28 | 10856 | RUVBL2 | RuvB-like 2 (E. coli) | 0.00E+00 | 386 | 184 | 8.55 | -11.67 | 11.67 | - |
| 29 | 64764 | CREB3L2 | cAMP responsive element binding protein 3-like 2 | 0.00E+00 | 375 | 114 | 5.41 | 8.27 | 8.27 | + |
| 30 | 4088 | SMAD3 | SMAD family member 3 | 0.00E+00 | 374 | 133 | 10.83 | -9.86 | 9.86 | - |
| 31 | 90 | ACVR1 | activin A receptor, type I | 0.00E+00 | 373 | 161 | 9.69 | 10.70 | 10.70 | + |
| 32 | 29994 | BAZ2B | bromodomain adjacent to zinc finger domain, 2B | 0.00E+00 | 370 | 178 | 5.53 | 10.10 | 10.10 | + |
| 33 | 2114 | ETS2 | v-ets erythroblastosis virus E26 oncogene homolog 2 (avian) | 0.00E+00 | 370 | 72 | 5.22 | 6.05 | 6.05 | + |
| 34 | 10657 | KHDRBS1 | KH domain containing, RNA binding, signal transduction associated 1 | 0.00E+00 | 369 | 148 | 19.72 | -8.74 | 8.74 | - |
| 35 | 10362 | HMG20B | high mobility group 20B | 0.00E+00 | 368 | 67 | 1.32 | -3.34 | 3.34 | - |
| 36 | 7088 | TLE1 | transducin-like enhancer of split 1 (E(sp1) homolog, Drosophila) | 0.00E+00 | 368 | 150 | 6.15 | 11.04 | 11.04 | + |
| 37 | 9232 | PTTG1 | pituitary tumor-transforming 1 | 0.00E+00 | 364 | 180 | 10.12 | -12.19 | 12.19 | - |
| 38 | 3725 | JUN | jun proto-oncogene | 0.00E+00 | 363 | 59 | 13.09 | 5.38 | 5.38 | + |
| 39 | 1649 | DDIT3 | DNA-damage-inducible transcript 3 | 0.00E+00 | 361 | 156 | 8.97 | 10.06 | 10.06 | + |
| 40 | 3149 | HMGB3 | high mobility group box 3 | 0.00E+00 | 361 | 162 | 16.41 | -11.34 | 11.34 | - |
| 41 | 56731 | SLC2A4RG | SLC2A4 regulator | 6.00E-03 | 360 | 38 | 0.98 | -3.13 | 3.13 | - |
| 42 | 10520 | ZNF211 | zinc finger protein 211 | 0.00E+00 | 359 | 203 | 6.52 | 10.81 | 10.81 | + |
| 43 | 6595 | SMARCA2 | SWI/SNF related, matrix associated, actin dependent regulator of chromatin, subfamily a, member 2 | 0.00E+00 | 358 | 149 | 7.48 | -8.06 | 8.06 | - |
| 44 | 7743 | ZNF189 | zinc finger protein 189 | 0.00E+00 | 358 | 203 | 7.83 | 8.86 | 8.86 | + |
| 45 | 10608 | MXD4 | MAX dimerization protein 4 | 0.00E+00 | 356 | 170 | 5.69 | 10.42 | 10.42 | + |
| 46 | 8914 | TIMELESS | timeless homolog (Drosophila) | 0.00E+00 | 356 | 197 | 10.64 | -12.38 | 12.38 | - |
| 47 | 51317 | PHF21A | PHD finger protein 21A | 0.00E+00 | 353 | 204 | 6.40 | 11.10 | 11.10 | + |
| 48 | 221037 | JMJD1C | jumonji domain containing 1C | 0.00E+00 | 350 | 116 | 19.28 | 9.36 | 9.36 | + |
| 49 | 7334 | UBE2N | ubiquitin-conjugating enzyme E2N | 0.00E+00 | 350 | 108 | 7.72 | -9.13 | 9.13 | - |
| 50 | 55758 | RCOR3 | REST corepressor 3 | 0.00E+00 | 347 | 202 | 8.52 | 11.14 | 11.14 | + |
| 51 | 22906 | TRAK1 | trafficking protein, kinesin binding 1 | 0.00E+00 | 347 | 102 | 5.01 | 6.24 | 6.24 | + |
| 52 | 8878 | SQSTM1 | sequestosome 1 | 0.00E+00 | 345 | 111 | 11.53 | 8.35 | 8.35 | + |
| 53 | 467 | ATF3 | activating transcription factor 3 | 0.00E+00 | 341 | 109 | 16.25 | 8.38 | 8.38 | + |
| 54 | 2975 | GTF3C1 | general transcription factor IIIC, polypeptide 1, alpha 220kDa | 0.00E+00 | 341 | 110 | 2.37 | 5.04 | 5.04 | + |
| 55 | 4646 | MYO6 | myosin VI | 0.00E+00 | 341 | 138 | 3.40 | 7.42 | 7.42 | + |
| 56 | 6773 | STAT2 | signal transducer and activator of transcription 2, 113kDa | 0.00E+00 | 340 | 151 | 7.59 | 9.69 | 9.69 | + |
| 57 | 3727 | JUND | jun D proto-oncogene | 0.00E+00 | 337 | 152 | 8.61 | 10.06 | 10.06 | + |
| 58 | 9202 | ZMYM4 | zinc finger, MYM-type 4 | 0.00E+00 | 334 | 135 | 7.97 | 8.02 | 8.02 | + |
| 59 | 64288 | ZNF323 | zinc finger protein 323 | 0.00E+00 | 334 | 122 | 7.58 | 7.68 | 7.68 | + |
| 60 | 323 | APBB2 | amyloid beta (A4) precursor protein-binding, family B, member 2 | 0.00E+00 | 332 | 98 | 4.89 | 7.57 | 7.57 | + |
| 61 | 1810 | DR1 | down-regulator of transcription 1, TBP-binding (negative cofactor 2) | 0.00E+00 | 331 | 75 | 5.05 | -6.57 | 6.57 | - |
| 62 | 57862 | ZNF410 | zinc finger protein 410 | 0.00E+00 | 331 | 107 | 4.83 | 7.06 | 7.06 | + |
| 63 | 9611 | NCOR1 | nuclear receptor corepressor 1 | 0.00E+00 | 330 | 171 | 7.07 | 9.34 | 9.34 | + |
| 64 | 4221 | MEN1 | multiple endocrine neoplasia I | 0.00E+00 | 329 | 69 | 1.31 | 3.50 | 3.50 | + |
| 65 | 10745 | PHTF1 | putative homeodomain transcription factor 1 | 5.00E-03 | 328 | 54 | 1.08 | 3.06 | 3.06 | + |
| 66 | 10488 | CREB3 | cAMP responsive element binding protein 3 | 0.00E+00 | 327 | 121 | 8.98 | 8.00 | 8.00 | + |
| 67 | 5087 | PBX1 | pre-B-cell leukemia homeobox 1 | 0.00E+00 | 326 | 59 | 4.41 | -5.94 | 5.94 | - |
| 68 | 7227 | TRPS1 | trichorhinophalangeal syndrome I | 0.00E+00 | 325 | 157 | 12.95 | 9.39 | 9.39 | + |
| 69 | 7494 | XBP1 | X-box binding protein 1 | 0.00E+00 | 325 | 102 | 14.17 | 7.55 | 7.55 | + |
| 70 | 1051 | CEBPB | CCAAT/enhancer binding protein (C/EBP), beta | 0.00E+00 | 323 | 106 | 6.89 | 8.07 | 8.07 | + |
| 71 | 9907 | KIAA0415 | KIAA0415 | 0.00E+00 | 322 | 147 | 7.54 | 9.17 | 9.17 | + |
| 72 | 54585 | LZTFL1 | leucine zipper transcription factor-like 1 | 0.00E+00 | 322 | 185 | 6.67 | 10.57 | 10.57 | + |
| 73 | 7020 | TFAP2A | transcription factor AP-2 alpha (activating enhancer binding protein 2 alpha) | 0.00E+00 | 322 | 36 | 5.16 | 6.01 | 6.01 | + |
| 74 | 22890 | ZBTB1 | zinc finger and BTB domain containing 1 | 0.00E+00 | 319 | 105 | 6.41 | 7.47 | 7.47 | + |
| 75 | 3397 | ID1 | inhibitor of DNA binding 1, dominant negative helix-loop-helix protein | 0.00E+00 | 318 | 59 | 6.94 | 5.64 | 5.64 | + |
| 76 | 5245 | PHB | prohibitin | 0.00E+00 | 318 | 149 | 7.79 | -9.44 | 9.44 | - |
| 77 | 3720 | JARID2 | jumonji, AT rich interactive domain 2 | 0.00E+00 | 317 | 92 | 3.91 | 7.57 | 7.57 | + |
| 78 | 7637 | ZNF84 | zinc finger protein 84 | 0.00E+00 | 316 | 150 | 6.19 | 8.83 | 8.83 | + |
| 79 | 9139 | CBFA2T2 | core-binding factor, runt domain, alpha subunit 2; translocated to, 2 | 0.00E+00 | 315 | 205 | 7.97 | 10.69 | 10.69 | + |
| 80 | 10935 | PRDX3 | peroxiredoxin 3 | 0.00E+00 | 314 | 175 | 9.81 | -10.72 | 10.72 | - |
| 81 | 64215 | DNAJC1 | DnaJ (Hsp40) homolog, subfamily C, member 1 | 0.00E+00 | 313 | 74 | 13.01 | 7.14 | 7.14 | + |
| 82 | 4205 | MEF2A | myocyte enhancer factor 2A | 0.00E+00 | 312 | 162 | 7.68 | 8.90 | 8.90 | + |
| 83 | 51274 | KLF3 | Kruppel-like factor 3 (basic) | 0.00E+00 | 311 | 164 | 7.42 | 10.34 | 10.34 | + |
| 84 | 7027 | TFDP1 | transcription factor Dp-1 | 0.00E+00 | 310 | 161 | 15.56 | -11.73 | 11.73 | - |
| 85 | 79230 | ZNF557 | zinc finger protein 557 | 0.00E+00 | 310 | 197 | 6.66 | 9.68 | 9.68 | + |
| 86 | 10379 | IRF9 | interferon regulatory factor 9 | 0.00E+00 | 309 | 140 | 11.25 | 9.18 | 9.18 | + |
| 87 | 25897 | RNF19A | ring finger protein 19A | 0.00E+00 | 305 | 139 | 9.51 | 9.14 | 9.14 | + |
| 88 | 6672 | SP100 | SP100 nuclear antigen | 0.00E+00 | 304 | 159 | 8.48 | 9.29 | 9.29 | + |
| 89 | 4084 | MXD1 | MAX dimerization protein 1 | 0.00E+00 | 303 | 145 | 9.79 | 9.68 | 9.68 | + |
| 90 | 1997 | ELF1 | E74-like factor 1 (ets domain transcription factor) | 0.00E+00 | 301 | 113 | 10.41 | 7.70 | 7.70 | + |
| 91 | 2130 | EWSR1 | Ewing sarcoma breakpoint region 1 | 0.00E+00 | 301 | 93 | 12.31 | -8.79 | 8.79 | - |
| 92 | 26959 | HBP1 | HMG-box transcription factor 1 | 0.00E+00 | 301 | 137 | 7.56 | 9.51 | 9.51 | + |
| 93 | 688 | KLF5 | Kruppel-like factor 5 (intestinal) | 0.00E+00 | 301 | 42 | 5.83 | 5.71 | 5.71 | + |
| 94 | 10849 | CD3EAP | CD3e molecule, epsilon associated protein | 0.00E+00 | 300 | 157 | 17.60 | -11.50 | 11.50 | - |
| 95 | 1993 | ELAVL2 | ELAV (embryonic lethal, abnormal vision, Drosophila)-like 2 (Hu antigen B) | 0.00E+00 | 299 | 64 | 8.53 | -5.82 | 5.82 | - |
| 96 | 10127 | ZNF263 | zinc finger protein 263 | 0.00E+00 | 299 | 163 | 6.95 | 9.02 | 9.02 | + |
| 97 | 7913 | DEK | DEK oncogene | 0.00E+00 | 298 | 95 | 8.99 | -7.58 | 7.58 | - |
| 98 | 55634 | ZNF673 | zinc finger family member 673 | 0.00E+00 | 298 | 106 | 4.75 | 7.14 | 7.14 | + |
| 99 | 7626 | ZNF75D | zinc finger protein 75D | 0.00E+00 | 298 | 185 | 9.31 | 8.85 | 8.85 | + |
| 100 | 4303 | FOXO4 | forkhead box O4 | 0.00E+00 | 297 | 104 | 7.01 | 7.73 | 7.73 | + |
| 101 | 9774 | BCLAF1 | BCL2-associated transcription factor 1 | 0.00E+00 | 295 | 84 | 4.46 | -6.51 | 6.51 | - |
| 102 | 406 | ARNTL | aryl hydrocarbon receptor nuclear translocator-like | 0.00E+00 | 293 | 182 | 10.90 | 10.44 | 10.44 | + |
| 103 | 9735 | KNTC1 | kinetochore associated 1 | 0.00E+00 | 290 | 161 | 10.77 | -10.71 | 10.71 | - |
| 104 | 7289 | TULP3 | tubby like protein 3 | 0.00E+00 | 290 | 96 | 8.69 | 6.50 | 6.50 | + |
| 105 | 2355 | FOSL2 | FOS-like antigen 2 | 0.00E+00 | 289 | 86 | 4.46 | 7.28 | 7.28 | + |
| 106 | 7756 | ZNF207 | zinc finger protein 207 | 0.00E+00 | 288 | 84 | 5.82 | -7.81 | 7.81 | - |
| 107 | 57798 | GATAD1 | GATA zinc finger domain containing 1 | 0.00E+00 | 283 | 147 | 10.34 | 8.76 | 8.76 | + |
| 108 | 57178 | ZMIZ1 | zinc finger, MIZ-type containing 1 | 0.00E+00 | 282 | 74 | 4.44 | 7.61 | 7.61 | + |
| 109 | 5036 | PA2G4 | proliferation-associated 2G4, 38kDa | 0.00E+00 | 281 | 179 | 14.26 | -11.18 | 11.18 | - |
| 110 | 8462 | KLF11 | Kruppel-like factor 11 | 0.00E+00 | 280 | 40 | 4.82 | 5.70 | 5.70 | + |
| 111 | 4869 | NPM1 | nucleophosmin (nucleolar phosphoprotein B23, numatrin) | 0.00E+00 | 280 | 151 | 9.74 | -8.03 | 8.03 | - |
| 112 | 79149 | ZSCAN5A | zinc finger and SCAN domain containing 5A | 0.00E+00 | 278 | 74 | 3.93 | 5.53 | 5.53 | + |
| 113 | 22926 | ATF6 | activating transcription factor 6 | 0.00E+00 | 277 | 131 | 8.89 | 8.49 | 8.49 | + |
| 114 | 80345 | ZSCAN16 | zinc finger and SCAN domain containing 16 | 0.00E+00 | 277 | 124 | 6.08 | 8.07 | 8.07 | + |
| 115 | 6662 | SOX9 | SRY (sex determining region Y)-box 9 | 1.00E-03 | 276 | 32 | 4.51 | -3.76 | 3.76 | - |
| 116 | 6829 | SUPT5H | suppressor of Ty 5 homolog (S. cerevisiae) | 0.00E+00 | 276 | 146 | 6.42 | 8.37 | 8.37 | + |
| 117 | 11108 | PRDM4 | PR domain containing 4 | 0.00E+00 | 275 | 116 | 8.66 | 8.02 | 8.02 | + |
| 118 | 8216 | LZTR1 | leucine-zipper-like transcription regulator 1 | 0.00E+00 | 273 | 107 | 4.92 | 7.35 | 7.35 | + |
| 119 | 1054 | CEBPG | CCAAT/enhancer binding protein (C/EBP), gamma | 0.00E+00 | 272 | 149 | 9.61 | 9.06 | 9.06 | + |
| 120 | 56995 | TULP4 | tubby like protein 4 | 0.00E+00 | 272 | 63 | 5.88 | 5.80 | 5.80 | + |
| 121 | 65986 | ZBTB10 | zinc finger and BTB domain containing 10 | 0.00E+00 | 272 | 129 | 6.27 | 9.18 | 9.18 | + |
| 122 | 2115 | ETV1 | ets variant 1 | 0.00E+00 | 271 | 29 | 3.55 | -3.61 | 3.61 | - |
| 123 | 81856 | ZNF611 | zinc finger protein 611 | 0.00E+00 | 271 | 82 | 7.34 | 5.69 | 5.69 | + |
| 124 | 9314 | KLF4 | Kruppel-like factor 4 (gut) | 0.00E+00 | 270 | 73 | 8.19 | 6.83 | 6.83 | + |
| 125 | 51621 | KLF13 | Kruppel-like factor 13 | 0.00E+00 | 268 | 113 | 6.29 | 7.68 | 7.68 | + |
| 126 | 4603 | MYBL1 | v-myb myeloblastosis viral oncogene homolog (avian)-like 1 | 0.00E+00 | 268 | 144 | 11.84 | -10.73 | 10.73 | - |
| 127 | 8061 | FOSL1 | FOS-like antigen 1 | 0.00E+00 | 267 | 51 | 3.13 | -4.60 | 4.60 | - |
| 128 | 4091 | SMAD6 | SMAD family member 6 | 0.00E+00 | 267 | 58 | 6.84 | -6.61 | 6.61 | - |
| 129 | 4780 | NFE2L2 | nuclear factor (erythroid-derived 2)-like 2 | 0.00E+00 | 265 | 98 | 7.02 | 7.25 | 7.25 | + |
| 130 | 5325 | PLAGL1 | pleiomorphic adenoma gene-like 1 | 0.00E+00 | 265 | 30 | 6.11 | 4.47 | 4.47 | + |
| 131 | 9338 | TCEAL1 | transcription elongation factor A (SII)-like 1 | 0.00E+00 | 265 | 27 | 6.92 | 3.86 | 3.86 | + |
| 132 | 10771 | ZMYND11 | zinc finger, MYND-type containing 11 | 0.00E+00 | 265 | 116 | 7.11 | 7.51 | 7.51 | + |
| 133 | 3131 | HLF | hepatic leukemia factor | 0.00E+00 | 262 | 34 | 8.56 | -4.09 | 4.09 | - |
| 134 | 7025 | NR2F1 | nuclear receptor subfamily 2, group F, member 1 | 0.00E+00 | 261 | 42 | 4.85 | -5.40 | 5.40 | - |
| 135 | 4783 | NFIL3 | nuclear factor, interleukin 3 regulated | 0.00E+00 | 260 | 113 | 8.84 | 7.84 | 7.84 | + |
| 136 | 7555 | CNBP | CCHC-type zinc finger, nucleic acid binding protein | 0.00E+00 | 258 | 40 | 3.10 | -5.55 | 5.55 | - |
| 137 | 168544 | ZNF467 | zinc finger protein 467 | 0.00E+00 | 258 | 58 | 3.68 | 6.01 | 6.01 | + |
| 138 | 9031 | BAZ1B | bromodomain adjacent to zinc finger domain, 1B | 0.00E+00 | 257 | 98 | 6.08 | -6.65 | 6.65 | - |
| 139 | 25909 | AHCTF1 | AT hook containing transcription factor 1 | 0.00E+00 | 256 | 123 | 7.02 | -8.44 | 8.44 | - |
| 140 | 7718 | ZNF165 | zinc finger protein 165 | 0.00E+00 | 256 | 67 | 8.11 | 6.05 | 6.05 | + |
| 141 | 3096 | HIVEP1 | human immunodeficiency virus type I enhancer binding protein 1 | 0.00E+00 | 254 | 88 | 7.88 | 7.29 | 7.29 | + |
| 142 | 22993 | HMGXB3 | HMG box domain containing 3 | 0.00E+00 | 253 | 101 | 10.08 | 6.88 | 6.88 | + |
| 143 | 9975 | NR1D2 | nuclear receptor subfamily 1, group D, member 2 | 0.00E+00 | 252 | 86 | 15.30 | 6.95 | 6.95 | + |
| 144 | 56987 | BBX | bobby sox homolog (Drosophila) | 0.00E+00 | 251 | 81 | 18.64 | 6.24 | 6.24 | + |
| 145 | 4929 | NR4A2 | nuclear receptor subfamily 4, group A, member 2 | 0.00E+00 | 251 | 100 | 5.27 | 7.51 | 7.51 | + |
| 146 | 7693 | ZNF134 | zinc finger protein 134 | 0.00E+00 | 251 | 130 | 7.99 | 7.89 | 7.89 | + |
| 147 | 79724 | ZNF768 | zinc finger protein 768 | 0.00E+00 | 251 | 116 | 6.00 | 7.43 | 7.43 | + |
| 148 | 3659 | IRF1 | interferon regulatory factor 1 | 0.00E+00 | 250 | 94 | 8.63 | 7.66 | 7.66 | + |
| 149 | 57209 | ZNF248 | zinc finger protein 248 | 3.00E-03 | 250 | 10 | 3.42 | -2.81 | 2.81 | - |
| 150 | 1831 | TSC22D3 | TSC22 domain family, member 3 | 0.00E+00 | 249 | 106 | 6.76 | 7.79 | 7.79 | + |
| 151 | 27107 | ZBTB11 | zinc finger and BTB domain containing 11 | 0.00E+00 | 249 | 68 | 9.40 | -5.27 | 5.27 | - |
| 152 | 23528 | ZNF281 | zinc finger protein 281 | 0.00E+00 | 249 | 64 | 9.84 | 5.73 | 5.73 | + |
| 153 | 4097 | MAFG | v-maf musculoaponeurotic fibrosarcoma oncogene homolog G (avian) | 0.00E+00 | 248 | 94 | 7.93 | 6.84 | 6.84 | + |
| 154 | 11198 | SUPT16H | suppressor of Ty 16 homolog (S. cerevisiae) | 0.00E+00 | 248 | 61 | 10.86 | -6.31 | 6.31 | - |
| 155 | 2959 | GTF2B | general transcription factor IIB | 0.00E+00 | 247 | 109 | 7.39 | 8.14 | 8.14 | + |
| 156 | 10725 | NFAT5 | nuclear factor of activated T-cells 5, tonicity-responsive | 0.00E+00 | 247 | 58 | 7.62 | 6.21 | 6.21 | + |
| 157 | 3726 | JUNB | jun B proto-oncogene | 0.00E+00 | 246 | 59 | 5.82 | 5.60 | 5.60 | + |
| 158 | 26009 | ZZZ3 | zinc finger, ZZ-type containing 3 | 0.00E+00 | 246 | 62 | 10.92 | 5.94 | 5.94 | + |
| 159 | 23764 | MAFF | v-maf musculoaponeurotic fibrosarcoma oncogene homolog F (avian) | 0.00E+00 | 244 | 41 | 7.77 | 6.20 | 6.20 | + |
| 160 | 4779 | NFE2L1 | nuclear factor (erythroid-derived 2)-like 1 | 0.00E+00 | 241 | 86 | 5.48 | 6.62 | 6.62 | + |
| 161 | 4801 | NFYB | nuclear transcription factor Y, beta | 0.00E+00 | 241 | 89 | 3.83 | -5.67 | 5.67 | - |
| 162 | 1827 | RCAN1 | regulator of calcineurin 1 | 0.00E+00 | 240 | 61 | 9.13 | 5.35 | 5.35 | + |
| 163 | 9569 | GTF2IRD1 | GTF2I repeat domain containing 1 | 0.00E+00 | 239 | 64 | 6.67 | 7.26 | 7.26 | + |
| 164 | 8609 | KLF7 | Kruppel-like factor 7 (ubiquitous) | 0.00E+00 | 239 | 67 | 3.30 | 6.03 | 6.03 | + |
| 165 | 10782 | ZNF274 | zinc finger protein 274 | 0.00E+00 | 239 | 92 | 8.34 | 7.16 | 7.16 | + |
| 166 | 5993 | RFX5 | regulatory factor X, 5 (influences HLA class II expression) | 0.00E+00 | 238 | 41 | 3.65 | 4.58 | 4.58 | + |
| 167 | 53335 | BCL11A | B-cell CLL/lymphoma 11A (zinc finger protein) | 0.00E+00 | 237 | 31 | 7.22 | -4.61 | 4.61 | - |
| 168 | 80095 | ZNF606 | zinc finger protein 606 | 0.00E+00 | 237 | 118 | 5.90 | 7.75 | 7.75 | + |
| 169 | 6772 | STAT1 | signal transducer and activator of transcription 1, 91kDa | 0.00E+00 | 236 | 49 | 6.51 | 5.83 | 5.83 | + |
| 170 | 5468 | PPARG | peroxisome proliferator-activated receptor gamma | 0.00E+00 | 235 | 31 | 7.08 | -4.54 | 4.54 | - |
| 171 | 4335 | MNT | MAX binding protein | 0.00E+00 | 234 | 108 | 4.18 | 7.79 | 7.79 | + |
| 172 | 1316 | KLF6 | Kruppel-like factor 6 | 0.00E+00 | 233 | 37 | 8.64 | 5.69 | 5.69 | + |
| 173 | 2908 | NR3C1 | nuclear receptor subfamily 3, group C, member 1 (glucocorticoid receptor) | 0.00E+00 | 232 | 117 | 10.57 | 8.04 | 8.04 | + |
| 174 | 654 | BMP6 | bone morphogenetic protein 6 | 0.00E+00 | 230 | 63 | 8.12 | 5.75 | 5.75 | + |
| 175 | 51710 | ZNF44 | zinc finger protein 44 | 0.00E+00 | 230 | 83 | 6.29 | 6.74 | 6.74 | + |
| 176 | 26137 | ZBTB20 | zinc finger and BTB domain containing 20 | 0.00E+00 | 228 | 130 | 7.03 | 9.28 | 9.28 | + |
| 177 | 2005 | ELK4 | ELK4, ETS-domain protein (SRF accessory protein 1) | 0.00E+00 | 227 | 71 | 5.80 | 5.53 | 5.53 | + |
| 178 | 9572 | NR1D1 | nuclear receptor subfamily 1, group D, member 1 | 0.00E+00 | 224 | 40 | 3.36 | 5.54 | 5.54 | + |
| 179 | 7157 | TP53 | tumor protein p53 | 1.00E-03 | 224 | 26 | 4.49 | 3.33 | 3.33 | + |
| 180 | 2637 | GBX2 | gastrulation brain homeobox 2 | 0.00E+00 | 223 | 80 | 12.24 | -7.44 | 7.44 | - |
| 181 | 9422 | ZNF264 | zinc finger protein 264 | 0.00E+00 | 223 | 108 | 5.87 | 7.00 | 7.00 | + |
| 182 | 6938 | TCF12 | transcription factor 12 | 0.00E+00 | 222 | 107 | 7.58 | 9.04 | 9.04 | + |
| 183 | 51276 | ZNF571 | zinc finger protein 571 | 0.00E+00 | 222 | 124 | 5.47 | 8.10 | 8.10 | + |
| 184 | 3223 | HOXC6 | homeobox C6 | 0.00E+00 | 221 | 54 | 5.53 | 5.59 | 5.59 | + |
| 185 | 23650 | TRIM29 | tripartite motif containing 29 | 0.00E+00 | 220 | 43 | 16.25 | 5.29 | 5.29 | + |
| 186 | 55713 | ZNF334 | zinc finger protein 334 | 0.00E+00 | 220 | 58 | 6.07 | 5.77 | 5.77 | + |
| 187 | 604 | BCL6 | B-cell CLL/lymphoma 6 | 0.00E+00 | 218 | 85 | 5.35 | 7.67 | 7.67 | + |
| 188 | 6778 | STAT6 | signal transducer and activator of transcription 6, interleukin-4 induced | 0.00E+00 | 218 | 64 | 8.27 | 5.60 | 5.60 | + |
| 189 | 55422 | ZNF331 | zinc finger protein 331 | 0.00E+00 | 218 | 78 | 7.53 | -5.22 | 5.22 | - |
| 190 | 22887 | FOXJ3 | forkhead box J3 | 0.00E+00 | 217 | 77 | 4.78 | 6.81 | 6.81 | + |
| 191 | 51385 | ZNF589 | zinc finger protein 589 | 0.00E+00 | 214 | 105 | 5.83 | 6.44 | 6.44 | + |
| 192 | 86 | ACTL6A | actin-like 6A | 0.00E+00 | 211 | 12 | 7.17 | -3.26 | 3.26 | - |
| 193 | 8880 | FUBP1 | far upstream element (FUSE) binding protein 1 | 7.00E-03 | 211 | 18 | 8.18 | -2.62 | 2.62 | - |
| 194 | 6940 | ZNF354A | zinc finger protein 354A | 0.00E+00 | 209 | 62 | 7.00 | 5.10 | 5.10 | + |
| 195 | 7050 | TGIF1 | TGFB-induced factor homeobox 1 | 0.00E+00 | 208 | 67 | 10.96 | 7.11 | 7.11 | + |
| 196 | 27125 | AFF4 | AF4/FMR2 family, member 4 | 0.00E+00 | 206 | 91 | 12.44 | 6.94 | 6.94 | + |
| 197 | 10923 | SUB1 | SUB1 homolog (S. cerevisiae) | 0.00E+00 | 206 | 82 | 5.31 | -7.78 | 7.78 | - |
| 198 | 26060 | APPL1 | adaptor protein, phosphotyrosine interaction, PH domain and leucine zipper containing 1 | 0.00E+00 | 204 | 37 | 11.35 | -4.20 | 4.20 | - |
| 199 | 57343 | ZNF304 | zinc finger protein 304 | 0.00E+00 | 203 | 87 | 10.60 | 6.33 | 6.33 | + |
| 200 | 3608 | ILF2 | interleukin enhancer binding factor 2, 45kDa | 0.00E+00 | 201 | 58 | 3.96 | -6.24 | 6.24 | - |
| 201 | 6493 | SIM2 | single-minded homolog 2 (Drosophila) | 0.00E+00 | 198 | 16 | 2.61 | 3.73 | 3.73 | + |
| 202 | 3665 | IRF7 | interferon regulatory factor 7 | 0.00E+00 | 197 | 60 | 7.32 | 5.61 | 5.61 | + |
| 203 | 23411 | SIRT1 | sirtuin 1 | 0.00E+00 | 196 | 45 | 19.64 | 5.44 | 5.44 | + |
| 204 | 50485 | SMARCAL1 | SWI/SNF related, matrix associated, actin dependent regulator of chromatin, subfamily a-like 1 | 0.00E+00 | 195 | 74 | 8.64 | 6.73 | 6.73 | + |
| 205 | 7748 | ZNF195 | zinc finger protein 195 | 0.00E+00 | 190 | 90 | 3.96 | 6.14 | 6.14 | + |
| 206 | 9988 | DMTF1 | cyclin D binding myb-like transcription factor 1 | 0.00E+00 | 188 | 69 | 7.76 | 5.91 | 5.91 | + |
| 207 | 23435 | TARDBP | TAR DNA binding protein | 0.00E+00 | 185 | 23 | 8.76 | -3.96 | 3.96 | - |
| 208 | 6925 | TCF4 | transcription factor 4 | 1.00E-03 | 182 | 35 | 29.04 | -3.39 | 3.39 | - |
| 209 | 3399 | ID3 | inhibitor of DNA binding 3, dominant negative helix-loop-helix protein | 0.00E+00 | 178 | 15 | 12.94 | 3.36 | 3.36 | + |
| 210 | 65982 | ZSCAN18 | zinc finger and SCAN domain containing 18 | 0.00E+00 | 175 | 22 | 4.98 | 4.06 | 4.06 | + |
| 211 | 7013 | TERF1 | telomeric repeat binding factor (NIMA-interacting) 1 | 0.00E+00 | 172 | 30 | 6.40 | -4.76 | 4.76 | - |
| 212 | 7767 | ZNF224 | zinc finger protein 224 | 0.00E+00 | 172 | 107 | 6.95 | 7.40 | 7.40 | + |
| 213 | 7571 | ZNF23 | zinc finger protein 23 (KOX 16) | 0.00E+00 | 171 | 77 | 7.42 | 6.63 | 6.63 | + |
| 214 | 90333 | ZNF468 | zinc finger protein 468 | 0.00E+00 | 166 | 60 | 6.75 | 6.27 | 6.27 | + |
| 215 | 6934 | TCF7L2 | transcription factor 7-like 2 (T-cell specific, HMG-box) | 0.00E+00 | 163 | 28 | 3.12 | -4.48 | 4.48 | - |
| 216 | 79673 | ZNF329 | zinc finger protein 329 | 0.00E+00 | 163 | 91 | 8.31 | 7.16 | 7.16 | + |
| 217 | 3202 | HOXA5 | homeobox A5 | 1.00E-03 | 157 | 13 | 8.94 | 3.46 | 3.46 | + |
| 218 | 7342 | UBP1 | upstream binding protein 1 (LBP-1a) | 0.00E+00 | 156 | 24 | 7.65 | -3.46 | 3.46 | - |
| 219 | 93349 | SP140L | SP140 nuclear body protein-like | 0.00E+00 | 141 | 32 | 3.63 | 3.86 | 3.86 | + |
| 220 | 84124 | ZNF394 | zinc finger protein 394 | 0.00E+00 | 140 | 56 | 3.89 | 4.28 | 4.28 | + |
| 221 | 4092 | SMAD7 | SMAD family member 7 | 0.00E+00 | 138 | 38 | 8.58 | 4.65 | 4.65 | + |
| 222 | 22980 | TCF25 | transcription factor 25 (basic helix-loop-helix) | 1.00E-03 | 135 | 25 | 5.31 | 3.36 | 3.36 | + |
| 223 | 2353 | FOS | FBJ murine osteosarcoma viral oncogene homolog | 0.00E+00 | 133 | 17 | 4.28 | 3.40 | 3.40 | + |
| 224 | 689 | BTF3 | basic transcription factor 3 | 0.00E+00 | 119 | 26 | 4.79 | -3.83 | 3.83 | - |
| Note: The values of Odd Ratio, NES, and absolute NES were rounded off to two decimal places. The table is sorted largest to smallest by Markers in regulon.  ^a^ Markers in regulon, the number of markers (genes) found to be first neighbors of the master regulator in the loaded network.  ^b^ Number Leading Edge, the number markers (genes) which belong to the GSEA leading edge set.  ^c^ Odd Ratio, odds of a regulon gene being in the GSEA leading edge set / odds of a regulon gene being in the GSEA trailing edge set  ^d^ NES, GSEA normalized enrichment score for the regulon of the TF.  ^e^ Plus mode means that the MR is positively correlated with up-regulated regulons in the geraniol-cluster, whereas minus mode denotes that the MR is positively correlated with down-regulated genes. | | | | | | | | | | |

| **Table S6**. List of 58 target genes of E2F8. | | | | | | |
| --- | --- | --- | --- | --- | --- | --- |
| No. | Gene ID | Gene symbol | Gene description | -log_10_(*p*-value) * sign (*t*-value) ^a^ | Fold change ^b^ | *q-*value ^b^ |
| 1 | 991 | CDC20 | cell division cycle 20 homolog (S. cerevisiae) | -6.17 | 0.61 | 0.00 |
| 2 | 1063 | CENPF | centromere protein F, 350/400kDa (mitosin) | -6.15 | 0.56 | 0.00 |
| 3 | **701** | **BUB1B** | **budding uninhibited by benzimidazoles 1 homolog beta (yeast)** | **-5.94** | **0.61** | **0.00** |
| 4 | **890** | **CCNA2** | **cyclin A2** | **-5.92** | **0.64** | **0.00** |
| 5 | 4171 | MCM2 | minichromosome maintenance complex component 2 | -5.88 | 0.57 | 0.00 |
| 6 | 8318 | CDC45 | cell division cycle 45 homolog (S. cerevisiae) | -5.41 | 0.69 | 0.00 |
| 7 | **11113** | **CIT** | **citron (rho-interacting, serine/threonine kinase 21)** | **-5.23** | **0.67** | **0.00** |
| 8 | **23397** | **NCAPH** | **non-SMC condensin I complex, subunit H** | **-5.16** | **0.69** | **0.00** |
| 9 | 1033 | CDKN3 | cyclin-dependent kinase inhibitor 3 | -4.84 | 0.58 | 0.00 |
| 10 | **699** | **BUB1** | **budding uninhibited by benzimidazoles 1 homolog (yeast)** | **-4.81** | **0.71** | **0.00** |
| 11 | 6502 | SKP2 | S-phase kinase-associated protein 2 (p45) | -4.64 | 0.73 | 0.00 |
| 12 | **11004** | **KIF2C** | **kinesin family member 2C** | **-4.62** | **0.63** | **0.00** |
| 13 | **3835** | **KIF22** | **kinesin family member 22** | **-4.60** | **0.75** | **0.00** |
| 14 | 81620 | CDT1 | chromatin licensing and DNA replication factor 1 | -4.54 | 0.74 | 0.00 |
| 15 | 26271 | FBXO5 | F-box protein 5 | -4.40 | 0.71 | 0.00 |
| 16 | **51203** | **NUSAP1** | **nucleolar and spindle associated protein 1** | **-4.35** | **0.50** | **0.00** |
| 17 | 4678 | NASP | nuclear autoantigenic sperm protein (histone-binding) | -4.21 | 0.77 | 0.00 |
| 18 | 3832 | KIF11 | kinesin family member 11 | -4.19 | 0.63 | 0.00 |
| 19 | 1062 | CENPE | centromere protein E, 312kDa | -4.18 | 0.67 | 0.00 |
| 20 | 8317 | CDC7 | cell division cycle 7 homolog (S. cerevisiae) | -4.09 | 0.66 | 0.00 |
| 21 | 7272 | TTK | TTK protein kinase | -4.09 | 0.59 | 0.00 |
| 22 | **5888** | **RAD51** | **RAD51 homolog (S. cerevisiae)** | **-4.08** | **0.82** | **0.00** |
| 23 | 11065 | UBE2C | ubiquitin-conjugating enzyme E2C | -4.08 | 0.53 | 0.00 |
| 24 | **4751** | **NEK2** | **NIMA (never in mitosis gene a)-related kinase 2** | **-4.06** | **0.75** | **0.00** |
| 25 | 1017 | CDK2 | cyclin-dependent kinase 2 | -3.97 | 0.78 | 0.00 |
| 26 | 9700 | ESPL1 | extra spindle pole bodies homolog 1 (S. cerevisiae) | -3.96 | 0.68 | 0.00 |
| 27 | 672 | BRCA1 | breast cancer 1, early onset | -3.94 | 0.76 | 0.00 |
| 28 | 29127 | RACGAP1 | Rac GTPase activating protein 1 | -3.90 | 0.57 | 0.00 |
| 29 | **9088** | **PKMYT1** | **protein kinase, membrane associated tyrosine/threonine 1** | **-3.70** | **0.78** | **0.00** |
| 30 | 22974 | TPX2 | TPX2, microtubule-associated, homolog (Xenopus laevis) | -3.69 | 0.57 | 0.00 |
| 31 | **5347** | **PLK1** | **polo-like kinase 1** | **-3.68** | **0.67** | **0.00** |
| 32 | 1841 | DTYMK | deoxythymidylate kinase (thymidylate kinase) | -3.66 | 0.80 | 0.00 |
| 33 | **332** | **BIRC5** | **baculoviral IAP repeat containing 5** | **-3.64** | **0.65** | **0.00** |
| 34 | 5422 | POLA1 | polymerase (DNA directed), alpha 1, catalytic subunit | -3.62 | 0.72 | 0.00 |
| 35 | **56992** | **KIF15** | **kinesin family member 15** | **-3.61** | **0.63** | **0.00** |
| 36 | 5426 | POLE | polymerase (DNA directed), epsilon | -3.56 | 0.80 | 0.00 |
| 37 | 4288 | MKI67 | antigen identified by monoclonal antibody Ki-67 | -3.49 | 0.65 | 0.00 |
| 38 | 10051 | SMC4 | structural maintenance of chromosomes 4 | -3.24 | 0.57 | 0.00 |
| 39 | 51053 | GMNN | geminin, DNA replication inhibitor | -3.23 | 0.56 | 0.00 |
| 40 | 1031 | CDKN2C | cyclin-dependent kinase inhibitor 2C (p18, inhibits CDK4) | -3.21 | 0.70 | 0.00 |
| 41 | 9493 | KIF23 | kinesin family member 23 | -3.21 | 0.64 | 0.00 |
| 42 | **9735** | **KNTC1** | **kinetochore associated 1** | **-3.20** | **0.70** | **0.00** |
| 43 | **995** | **CDC25C** | **cell division cycle 25 homolog C (S. pombe)** | **-3.17** | **0.77** | **0.00** |
| 44 | 675 | BRCA2 | breast cancer 2, early onset | -3.06 | 0.84 | 0.00 |
| 45 | 2189 | FANCG | Fanconi anemia, complementation group G | -3.03 | 0.78 | 0.00 |
| 46 | 9055 | PRC1 | protein regulator of cytokinesis 1 | -3.00 | 0.53 | 0.00 |
| 47 | 993 | CDC25A | cell division cycle 25 homolog A (S. pombe) | -2.96 | 0.76 | 0.00 |
| 48 | 3925 | STMN1 | stathmin 1 | -2.87 | 0.82 | 0.00 |
| 49 | 4693 | NDP | Norrie disease (pseudoglioma) | -2.81 | 0.75 | 0.00 |
| 50 | **3306** | **HSPA2** | **heat shock 70kDa protein 2** | **-2.80** | **0.75** | **0.00** |
| 51 | 8438 | RAD54L | RAD54-like (S. cerevisiae) | -2.78 | 0.69 | 0.00 |
| 52 | 1111 | CHEK1 | CHK1 checkpoint homolog (S. pombe) | -2.76 | 0.75 | 0.00 |
| 53 | 10926 | DBF4 | DBF4 homolog (S. cerevisiae) | -2.60 | 0.75 | 0.00 |
| 54 | 25788 | RAD54B | RAD54 homolog B (S. cerevisiae) | -2.57 | 0.74 | 0.00 |
| 55 | 1869 | E2F1 | E2F transcription factor 1 | -2.55 | 0.81 | 0.00 |
| 56 | **4085** | **MAD2L1** | **MAD2 mitotic arrest deficient-like 1 (yeast)** | **-2.47** | **0.64** | **0.00** |
| 57 | 8914 | TIMELESS | timeless homolog (Drosophila) | -2.35 | 0.83 | 0.00 |
| 58 | 10293 | TRAIP | TRAF interacting protein | -1.71 | 0.89 | 0.11 |
| Note: The 58 target genes are sorted smallest to largest by -log_10_(*p-*value) * sign (*t-*value).  ^a^ A modified test statistic combining the -log_10_(*p-*value) with the sign of the *t-*value. The sign of the t-value indicates positive or negative differential expression.  ^b^ Fold change and *q-*value were calculated by SAM analysis. | | | | | | |
