# Supplementary material for: Geraniol suppresses prostate cancer growth through down‐regulation of E2F8
Source: Cancer Med. 2016 Sep 28;5(10):2899–908. doi: 10.1002/cam4.864 (PMC5083744; doi:10.1002/cam4.864)
Supplement: Supplementary file 13 [file CAM4-5-2899-s013.pdf]

KORAM BIOGEN CORP.

6F, KORAM VENTURE TOWN
907-1, DAECHI-DONG, KANGNAM-KU, SEOUL,
135-280, SOUTH KOREA

TEL:82(2)556-0311(Rep.)
FAX:82(2)556-0828 /9
Email: koram@ korambiotech.com

TO: Seoul National University, Min-A Seol

DATE : 1/28/2016

CC: Koramdeolab

RE: Purchase of ATCC Item

T.PAGES: 1

This letter is to confirm that Min-A Seol from Seoul National University received item CRL-1435 (non-GMO item) from ATCC.

The rest of their contact information is as follows:

Address: Yeongeon-dong, Jongno-gu, Seoul, Korea, 110-799
82-2-740-8217 phone
82-2-3673-2167 fax

The import declaration information is as follows:

ATCC product No.	AWB No.	Date
CRL-1435	774127335172	2015. 07. 27

Korambiogen corp.

CEO & President
